# Pharmacological treatments of Chinese herbal medicine for irritable bowel syndrome in adults: A network meta-analysis of randomized controlled trials

**DOI:** 10.1371/journal.pone.0255665

**Published:** 2021-08-06

**Authors:** Yun-bo Wu, Yun-kai Dai, Ling Zhang, Huai-geng Pan, Wei-jing Chen, Ru-liu Li, Ling Hu

**Affiliations:** 1 Institute of Gastroenterology, Guangzhou University of Chinese Medicine, Guangzhou, Guangdong, China; 2 Science and Technology Innovation Center, Guangzhou University of Chinese Medicine, Guangzhou, Guangdong, China; Goethe University Frankfurt: Goethe-Universitat Frankfurt am Main, GERMANY

## Abstract

**Introduction:**

Plenty of clinical studies have suggested the value of Chinese herbal medicine (CHM) for patients with irritable bowel syndrome (IBS), but their efficacy and safety have not been systematically concluded yet. This article aimed to compare and rank the therapeutic effect and safety of CHM with routine pharmacotherapies and placebo in the treatment of IBS.

**Methods:**

Randomized controlled trials regarding CHM to treat IBS were searched in six databases from inception to Jan 31, 2020. A network meta-analysis was conducted to analyze the data of included publications. The quality assessment was assessed by Cochrane Handbook and GRADEpro software. The risk ratio was calculated for dichotomous outcomes while the standardized mean difference was used for continuous variables with 95% credible intervals. A Funnel plot was performed to evaluate publication bias. The surface under the cumulative ranking curve was conducted to rank the included interventions. Data were analyzed with STATA 15.0 and Review Manager 5.3.

**Result:**

3194 records were searched, and 28 eligible trials involving 3323 patients ere identified. Compared with conventional therapies and placebo, Jianpi-Chushi therapy showed significant improvement in adequate relief and IBS symptom severity scale; Shugan-Jianpi therapy showed the best efficacy in relieving the abdominal pain and abdominal distension; Wenshen-Jianpi therapy had a better effect on avoiding adverse effects and improving stool character.

**Conclusion:**

This study confirmed that CHM could be beneficial for patients with IBS in relieving their clinical symptoms and should be recommended as alternative therapies. The quality of evidence in this study based on the GRADE system was “low”.

## Introduction

Irritable bowel disease (IBS) is one of the most common chronic digestive disorders in the world, which is characterized by abdominal pain and discomfort, defecation as well as change in stool consistency and frequency [1]. According to epidemiological research, the incidence ranges from 19.58%~23.40% in China and 10~25% in North America and Europe [2, 3]. According to the Rome IV criteria [4], IBS can be presented as 4 pattern subtypes: IBS with diarrhea (IBS-D), IBS with constipation (IBS-C), mixed IBS (IBS-M), and unclassified IBS (IBS-U).

The main pathogenesis of IBS has been conceptualized as a condition of visceral hypersensitivity (leading to abdominal discomfort or pain) [5], and gastrointestinal motor disturbances (leading to diarrhea or constipation) [6]. In addition, there is increasing evidence regarding the roles of mood and anxiety disorders, infection and immune activation, serotonin dysregulation, bacterial overgrowth, central dysregulation as well as brain-gut interaction, family genetics in the etiology of IBS [7–12]. Due to the diversity of pathogenesis, the main pharmacological treatments of IBS such as smooth-muscle relaxants, prokinetic agents, peripheral opioid agonist, antidiarrheal, antidepressants, and probiotics, can only achieve limited clinical benefits [13, 14], and some of them may even cause a risk of cardiovascular events in long term use [15]. Therefore, it is necessary to look for more effective and safer alternative therapies.

Traditional Chinese medicine (TCM) has been used to treat symptoms associated with IBS for thousands of years in East Asia and may offer insights into a more targeted approach for therapeutic development [16]. Plenty of previous studies have evaluated the efficacy and safety of CHM (Chinese herbal medicine) formulae in the treatment of IBS-C and IBS-D [17–20], but these studies focused on pairwise comparisons between single formula and conventional medicines, and no comparison with different CHM formulae was conducted in the treatment of IBS on a large scale.

Therefore, a Bayesian network meta-analysis (NMA) which integrates direct evidence with indirect for multiple intervention comparisons was performed to compare and rank different CHM formulae with routine pharmacotherapies in the management of clinical symptoms in patients with IBS.

## Methods

This study was performed in conformity to the Cochrane Handbook for the Systematic Review of Interventions and the Preferred Reporting Items for Systematic Review and Meta-Analyses [21]. The completed PRISMA checklist was presented as S1 File.

### Data source and search strategy

An electronic search was conducted in the following databases from their inception to January 31, 2020: PubMed, Springer, EMBASE, China National Knowledge Infrastructure, Chinese Biomedicine Database, and WanFang databases. The detailed search strategy for each database could be found in S2 File.

### Inclusion criteria and study selection

According to the PICOS (participants, interventions, comparisons, outcomes, and study design) criteria, inclusion criteria and exclusion criteria are summarized in Table 1. In the treatment group (participants in the treatment group should be treated by CHM used alone), CHM formulae, based on its function, were classified into 3 categories: soothe the liver and fortify the spleen (TCM jargon: Shugan-Jianpi) therapy (SJ), fortify the spleen and drain dampness (TCM jargon: Jianpi-Chushi) therapy (JC) and warm the kidney and fortify the spleen (TCM jargon: Wenshen-Jianpi) therapy (WJ). The formulations of CHM included decoction, tablet, pill, powder, granule, capsule, and oral liquid. The following interventions with usual care were included as the control group: placebo, antispasmodic agents (pinaverium and trimebutine), antidiarrheal (smectite), and probiotics.

**Table 1:** Eligibility criteria PICOs.

	Inclusion criteria	Exclusion criteria
Participants	Meet the diagnosis of Rome criteria of IBS	Patients under 18-year-old, patients with complication such as severe heart attack
Interventions	Pharmacological therapy of CHM (Shugan-Jianpi therapy, Jianpi-Chushi therapy, and Wenshen-Jianpi therapy)	
Comparisons	Routine pharmacological interventions (antispasmodic agents, antidiarrheal, probiotics, placebo)	
Outcomes	Primary outcome: adequate relief, IBS symptom severity scale; Secondary outcome: adverse effects; improvement of clinical symptoms.	
Study design	Randomized controlled trials; sample size >10/arm; Duration>4 weeks; Jadad score>2.	

IBS: Irritable Bowel Syndrome; CHM: Chinese herbal medicine.

### Data extraction and quality assessment

Two investigators independently selected the studies. The review of the selected studies, the extraction of the relevant information, and the assessment of the risk of bias tool were performed by two investigators. Relevant information was extracted from each included study: Study ID (first author and publication year), classification of disease and diagnostic criteria, the characteristics of participants (gender, age, and sample size), the course of disease, detailed of interventions (treatment and duration), primary outcomes (adequate relief, improvement of irritable bowel syndrome—severity scoring system (IBS-SSS)) and secondary outcomes (adverse effects, improvement of clinical symptoms). Any missing information will be acquired by contacting the corresponding author. The access to the included trials was displayed in S3 File.

The risk of bias of the included studies was evaluated with the Cochrane Collaboration Recommendations assessment tool [22]. Seven domains were assessed as low-risk, high-risk, or unclear-risk including random sequence generation, allocation concealment, blinding of participants and personnel, blinding (or) masking of outcomes assessors, incomplete outcome data, selective reporting, and other bias. Besides, the Grading of Recommendations Assessment, Development, and Evaluation (GRADE) was performed to assess the quality of evidence as high, moderate, low, or very low quality.

### Statistical analysis

An NMA with a Bayesian framework using Software for Statistics and Data Science (STATA, version 15.1) was conducted to assess the outcomes of different interventions. For continuous variables (IBS-SSS and the improvement of clinical symptoms), standardized mean differences (SMD) were calculated with a 95% confidence interval (CI). For dichotomous data (adequate relief, adverse effects), risk ratios (RR) were calculated with a 95% CI. Considering the diversity of interventions and potential heterogeneity among included studies, a random-effect model was applied in all meta-analyses. The consistency test results were judged by node-splitting analysis and an inconsistency model. When the p-value of the node-splitting analysis was greater than 0.05, a consistency mode was selected [23]; otherwise, an inconsistency model was used. Heterogeneity analysis was assessed through inconsistency index statistic (*I*^2^). The *I*^2^ value above 50% was considered as heterogeneity throughout the study. Additionally, sensitivity analysis was conducted to verify the robustness of the results and test the source of heterogeneity in each RCT. To summarize the probabilities for all interventions, the surface under the cumulative ranking curve (SUCRA) was selected to offer a summary statistic for the cumulative ranking [24]. Based on the definition, the larger SUCRA scores are, the more effective interventions are.

## Results

### Study identification and selection

**Fig 1:**
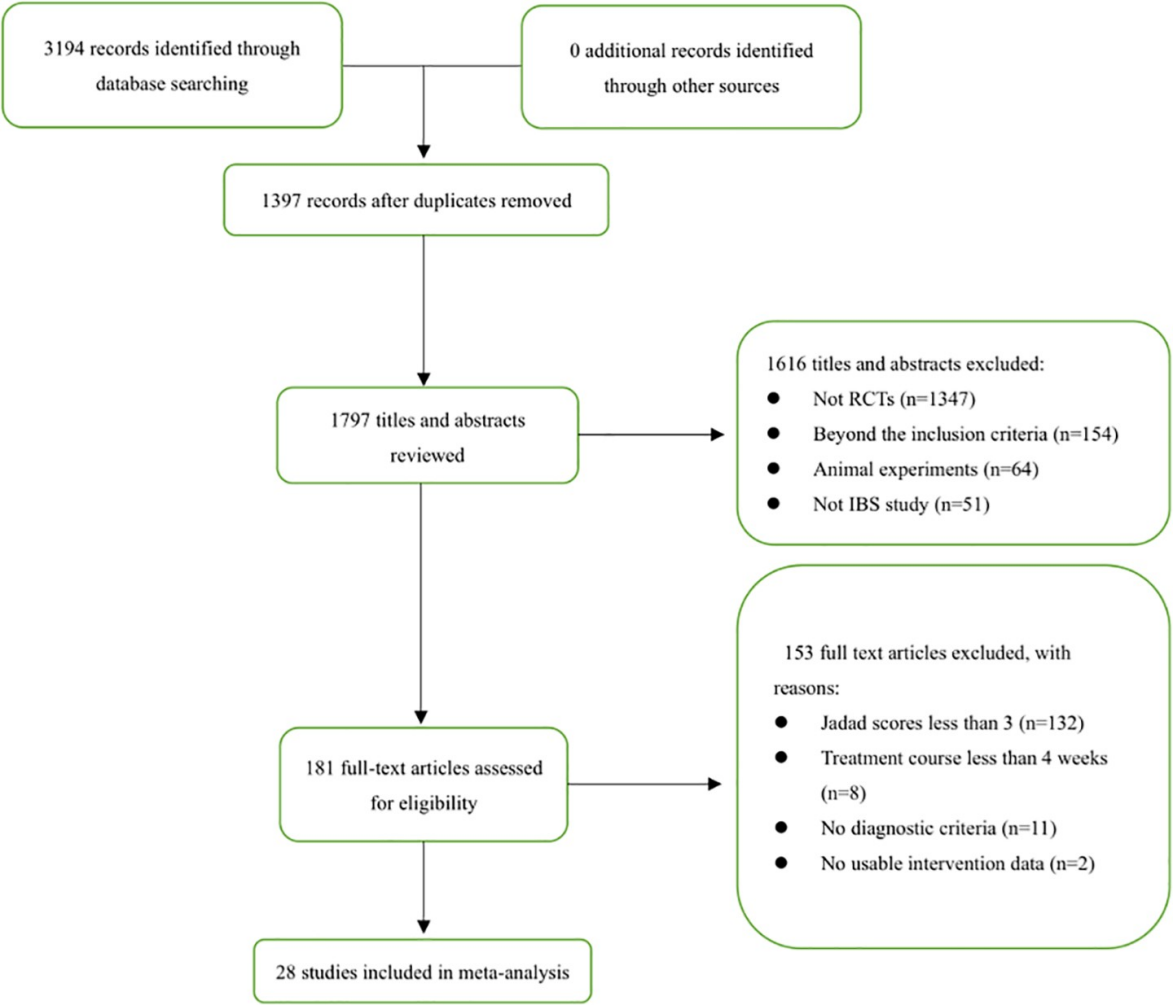
Flow diagram.

**Fig 2:**
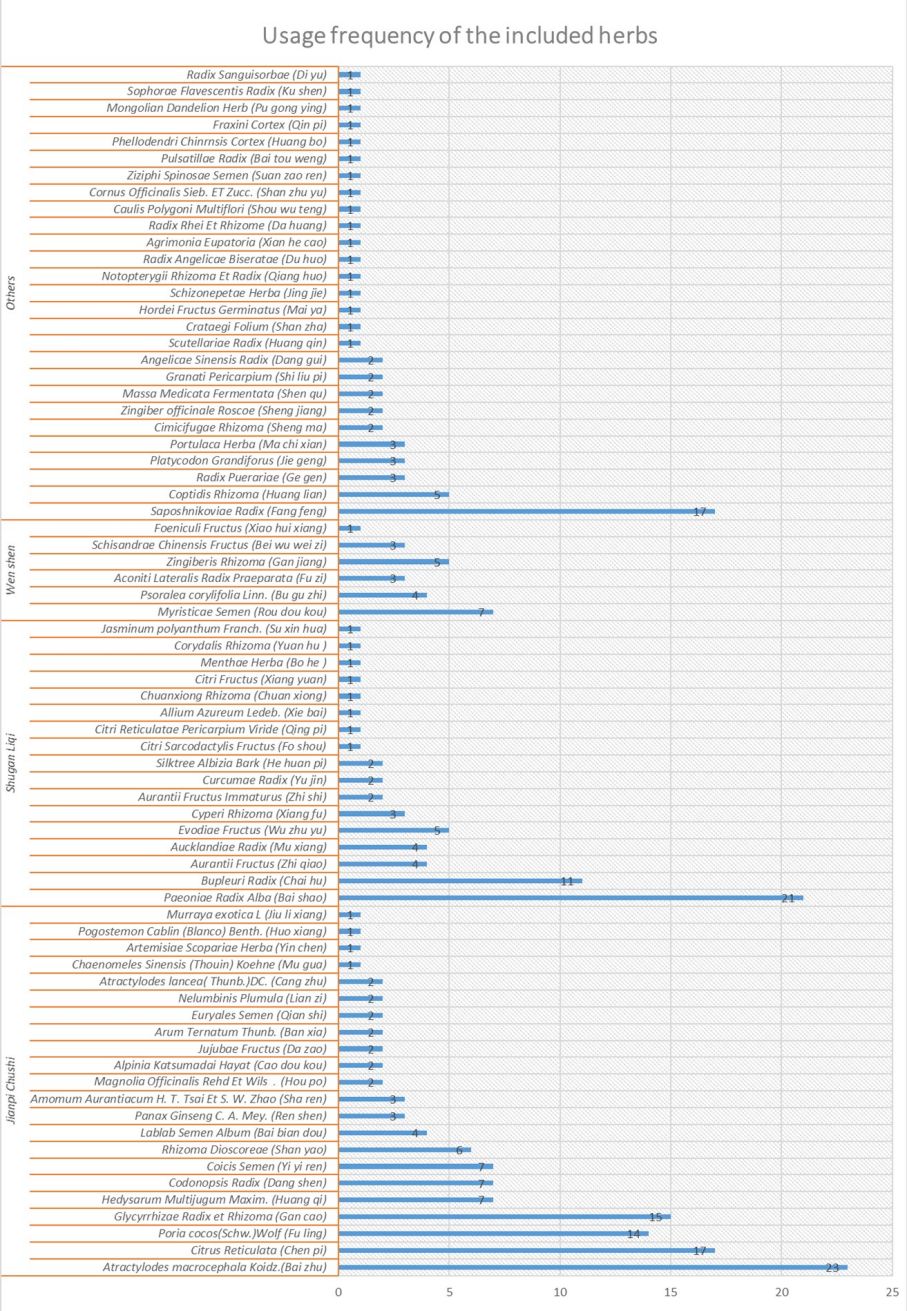
Usage frequency of the included herbs.

In total, 3194 citations (PubMed 43, Spring 643, EMBASE 73, CNKI 459, CBD 1933, WanFang 43) published from inception to January 31, 2020, were identified by the search. After removing duplicates and unrelated articles, 28 articles comprising 3323 patients were deemed eligible for further quantitative analyses [25–52]. A flow diagram of the specific screening procedures is shown in Fig 1. The baseline characteristics of the studies were extracted in Table 2. The frequency of utilization of the included herbs is summarized in Fig 2 while the components of each formula are summarized in Table 3.

**Table 2:** Characteristics of the studies included in the network analysis.

Study ID	Country	Classification of IBS	Diagnostic criteria	Sample Size		Study population	Age (years)	Course of disease (years)	Duration (weeks)	Intervention		Outcomes	Follow-up	Side effects
				EG (M/F)	CG (M/F)					EG	CG			
Chen 2019 [25]	China	IBS-D	Rome IV	13/16	14/16	Sigle center	E:37.97±11.63	N/A	4	SJ	Trimebutine	a, b, c, g, i	4 weeks	E:0/29
							C:37.50±10.80			100ml/b.i.d	0.1g/t.i.d			C:0/30
Shih et.al 2019 [26]	China	IBS-C/IBS-D	Rome III	11/21	9/22	Single center	E:43.07±13.77	N/A	4	SJ	Placebo	b, e, h, o, p	N/A	E:0/31
							C:43.96±12.49			3g/t.i.d				C:3/32
Tang et.al 2019 [27]	China	IBS-D	Rome III	85/86	93/78	Multi centers	E:43.97±13.82	E:2.02±1.92	6	SJ	Pinaverium Bromide 50mg/t.i.d	a, b, c, d, e, i	8 weeks	E:5/171
							C:45.59±12.81	C:2.16±2.94		5g/t.i.d				C:4/171
Wang 2019 [28]	China	IBS-D	Rome IV	13/17	17/15	Single center	E:38.57±13.44	E:4.47±3.51	8	JC	Pinaverium Bromide 50mg/t.i.d	a, b, c, f, g	6 months	E:0/30
							C:39.56±13.07	C:4.97±3.74		200ml/b.i.d				C:0/32
Yue 2019 [29]	China	IBS-D	Rome IV	11/16	13/10	Single center	E:32.48±8.00	E:4.29±2.54	8	JC	Trimebutine	a, b, c, d, e, f, g, h	8 weeks	E:0/27
							C:32.39±10.80	C:4.56±2.71			0.2g/t.i.d			C:0/23
Zhang 2019 [30]	China	IBS-D	Rome IV	18/17	14/21	Single center	E:46.40±10.31	N/A	4	SJ	Antidiarrheal	a, b, c, f, g	4 weeks	E:0/35
										200ml/t.i.d	3g/t.i.d			C:0/34
							C:44.40±10.08							
Zhao et.al 2019 [31]	China	IBS-D	Rome IV	18/13	17/12	Single center	E:34.3±5.0	E:3.2±0.6	4	JC	Pinaverium Bromide 50mg/t.i.d	a, b, e, l	N/A	E:5/31
							C:35.2±4.7	C:2.8±0.3		300ml/t.i.d				C:5/29
Zheng 2019 [32]	China	IBS-D	Rome IV	23/17	20/18	Single center	E:37.38±13.41	E:3.66±3.49	4	SJ	Antidiarrheal	a, b, c, d, f, g	2 months	E:1/40
							C:39.34±14.80	C:4.31±4.25		15oml/t.i.d	3g/t.i.d			C:2/38
Chen et.al 2018 [33]	China	IBS-D	Rome III	41/39	31/49	Multi centers	E:35.4±10.7	E:4.9±1.6	4	SJ 25.4g/t.i.d	Placebo	a, b, j	N/A	E:5/80
							C:32.7±8.2	C:5.4±1.5						C:4/80
Tang et.al 2018 [34]	China	IBS-D	Rome III	62/37	66/41	Multi centers	E:42.88±13.77	E:6.41±6.65	8	SJ 150ml/t.i.d	Placebo	a, b, d, e, k	N/A	E:5/99
							C:42.48±13.96	C:7.54±6.74						C:3/107
Fan et.al 2017 [35]	China	IBS-D	Rome III	146/202	154/194	Multi centers	E:36.3	E:5.48	4	SJ	Pinaverium Bromide 50mg/t.i.d	a, b, f, j	N/A	E:68/348
							C:36.5	C:5.9						C:65/348
Wang et.al 2017 [36]	China	IBS-D	Rome III	46/35	44/37	Single center	E:42.33±12.81	E:6.67±4.84		SJ	Trimebutine	a, f, l	N/A	N/A
							C:41.67±13.43	C:6.33±4.18		400ml/q.d	100mg/t.i.d			
Zhang 2017 [37]	China	IBS-D	Rome III	19/15	10/7	Single center	E:48.35±10.42	N/A	8	WJ	Trimebutine	a, b, c, d, e, g, h	8 weeks	E:1/34
							C:51.20±12.06							C:2/17
Chen 2016 [38]	China	IBS-D	Rome III	21/18	17/20	Single center	E:37.77±11.04	E:1.57±0.81	4	WJ	Pinaverium Bromide 50mg/t.i.d	a, b, e, g	N/A	E:0/39
							C:36.70±9.21	C:1.19±0.82		150ml/b.i.d				C:0/37
Huang et.al 2016 [39]	China	IBS-D	Rome III	20/25	18/24	Single center	E:42.70±6.53	E:9.25±3.53	4	SJ	Trimebutine	a, b, f	N/A	E:1/43
							C:41.32±5.72	C:8.65±3.74 months		150ml/b.i.d	100mg/t.i.d			C:1/40
Bensoussan et.al 2015 [40]	Australia	IBS-C	Rome III	4/57	5/59	Multi centers	N/A	N/A	8	SJ	Placebo	a, b, d, j, m	N/A	E:4/50
										4.2g/qd				C:2/58
Cheng 2015 [41]	China	IBS-D	Rome III	15/17	14/16	Single center	N/A	N/A	8	SJ	Trimebutine	a, b. d	N/A	E:0/32
										15oml/b.i.d	200mg/t.i.d			C:0/30
Huang 2015 [42]	China	IBS-D	Rome III	10/19	20/8	Single center	E:33.59±12.03	E:3.55±4.88	4	SJ	placebo	a, b, c, d, e	6 months	E:0/29
							C:36.96±13.26	C:3.98±4.83						C:0/28
Liang et.al 2015 [43]	China	IBS-D	Rome III	27/26	24/26	Single center	E:41	E:5.4	4	WJ	Pinaverium Bromide	a, b, f, q	N/A	E:0/52
							C:39	C:5.1		100ml/b.i.d	50mg/t.i.d			C:0/50
Wei 2015 [44]	China	IBS-D	Rome III	17/25	25/18	Single center	E:42.46±12.44	E:4.40±3.72	8	JC	Probiotics	a, b, g	N/A	E:2/42
							C:40.07±10.25	C:4.28±3.84		150ml/b.i.d	420mg/t.i.d			C:3/43
Yan 2015 [45]	China	IBS-D	Rome III	13/18	14/16	Single center	E:41.94±12.64	E:5.80±2.09	8	SJ	Trimebutine	a, b, d, g	N/A	E:0/31
							C:42.90±11.65	C:5.31±2.06		150ml/b.i.d	200mg/t.i.d			C:0/30
Chen et.al 2014 [46]	China	IBS-D	Rome III	38/20	32/26	Single center	E:38.48±11.93	E:5.81±5.04	4	SJ	Pinaverium Bromide	a, b, c, e, f, g, l, r	8 weeks	E:0/58
							C:38.35±11.75	C:5.90±4.12		150ml/b.i.d	50mg/t.i.d			E:0/58
Cai et.al 2013 [47]	China	IBS-D	Rome III	11/6	4/14	Single center	E:43.24±10.26	E:4.56±4.42	8	SJ	placebo	b, d, g	N/A	E:0/27
							C:41.89±9.33	C:4.98±5.01		150ml/t.i.d				C:0/28
Bian 2011 [48]	China	IBS-D	Rome III	19/9	12/18	Single center	E:47.68±12.98	E:6.65±8.64	4	WJ	placebo	a, b, d, e, g, k	N/A	E:5/38
							C:46.13±13.01	C:8.97±7.91		150ml/t.i.d				C:4/30
Liang et.al 2009 [49]	China	IBS-D	Rome III	7/13	9/11	Single center	E:38.30±7.83	E:6.15±2.90	4	SJ	Pinaverium Bromide	a, f, g	N/A	N/A
							C:38.75±5.91	C:6.95±2.30						
											50mg/t.i.d			
Wu 2009 [50]	China	IBS-D	Rome III	15/20	14/21	Single center	E:38.26±12.58	E:2.51±4.04	4	SJ	Probiotics	a, b, f	N/A	E:0/32
							C:37.00±11.12	C:2.94±4.03			0.42g/b.i.d			C:0/31
Zhao 2007 [51]	China	IBS-D	Rome II	44/25	3/29	Single center	E:37.10±10.40	E:1.7±0.3	4	SJ	Pinaverium	a, b, g	N/A	E:0/68
							C:36.90±8.90	C:1.5±0.2						C:1/66
											Bromide 50mg/t.i.d			
Leung et.al 2006 [52]	China	IBS-D	Rome II	31/29	26/33	Single center	E:45.4±11.9	N/A	8	SJ	placebo	a, b, g, n	N/A	E:2/60
							C:43.6±13.9							C:1/59

**Annotations:** E: experiment group; C: control group; N/A: not applicable; TCM: traditional Chinese medicine; M: male; F: female; IBS: Irritable Bowel Syndrome; IBS-D: diarrhea-predominant irritable bowel syndrome; IBS-C: Constipation-predominant irritable bowel syndrome; JC: Jianpi Chushi therapy; SJ: Shugan Jianpi therapy; WJ: Wenshen Jianpi therapy; a: overall efficiency; b: Adverse effect rate; c: Recurrent rate; d: IBS- symptom severity scale; e: IBS- Quality of life; f: Clinical symptoms scores; g: TCM symptom scores; h: the expression of Immunohistochemistry; i: Hamilton Anxiety Scale & Hamilton Depression Scale; j: Bristol Stool Form Scale; k: Hospital Anxiety and Depression Scale; l: IBS bowel symptom severity scale; m: IBS Visual Analogue Scale; n: SF-36; o: Total and specific scores of gastrointestinal symptom rating Scale; p: IBS-WHO-QOL; q: chronic liver disease questionnaire; r: IBS defecation state questionnaire.

**Table 3:** The ingredients of each formula in the included trials.

Author	Quality assessment (Y/N)	Ingredients of each formula			
**Chen 2019**	Y-National Food and Drug Administration National Drug Standards	*Atractylodes macrocephala Koidz*.	*Paeoniae Radix Alba*	*Bupleuri Radix*	*Citrus Reticulata*
		(Bai zhu) 12g	(Bai shao) 10g	(Chai hu) 10g	(Chen pi) 10g
		*Saposhnikoviae Radix*	*Codonopsis Radix*	*Rhizoma Dioscoreae*	*Poria cocos (Schw*.*) Wolf*
		(Fang feng) 10g	(Dang shen) 10g	(Shan yao) 10g	(Fu ling) 10g
		*Curcumae Radix*	*Glycyrrhizae Radix et Rhizoma* (Gan cao) 6g		
		(Yu jin) 10g			
**Shih et.al 2019**	Y- Brion Research Institute of Taiwan	*Aucklandiae Radix*	*Amomum Aurantiacum H*. *T*. *Tsai* Et S. W. Zhao	*Arum Ternatum Thunb*. (Ban xia) 2.5g	*Citrus Reticulata*
		(Mu xiang) 2g	(Sha ren) 2g		(Chen pi) 2g
		*Panax Ginseng C*. *A*. *Mey*.	*Poria cocos (Schw*.*) Wolf*	*Atractylodes macrocephala Koidz*.	*Glycyrrhizae Radix et Rhizoma* (Gan cao) 2g
		(Ren shen) 2.5g	(Fu ling) 5g	(Bai zhu) 5g	
		*Zingiber officinale Roscoe*			
		(Sheng jiang) 5g			
**Tang et.al 2019**	Y-National Food and Drug Administration National Drug Standards	*Paeoniae Radix Alba*	*Citri Reticulatae Pericarpium Viride*	*Allium Azureum Ledeb*.	*Atractylodes macrocephala Koidz*.
				(Xie bai)	
					(Bai zhu)
		(Bai shao)	(Qing pi)		
**Wang 2019**	Y-National Food and Drug Administration National Drug Standards	*Massa Medicata Fermentata*	*Crataegi Folium*	*Hordei Fructus Germinatus* (Mai ya) 10g	*Panax Ginseng C*. *A*. *Mey*.
		(Shen qu) 10g	(Shan zha) 10g		(Ren shen) 20g
		*Glycyrrhizae Radix et Rhizoma*	*Poria cocos (Schw*.*) Wolf*	*Citri Sarcodactylis Fructus*	*Citrus Reticulata*
		(Gan cao) 6	(Fu ling) 10g	(Fo shou) 6g	(Chen pi) 6g
		*Atractylodes macrocephala Koidz*.	*Saposhnikoviae Radix*		
		(Bai zhu) 10g	(Fang feng) 10g		
**Yue 2019**	Y-National Food and Drug Administration National Drug Standards	*Radix Puerariae*	*Coptidis Rhizoma*	*Scutellariae Radix*	*Glycyrrhizae Radix et Rhizoma*
		(Ge gen) 30g	(Huang lian) 10g	(Huang qin) 10g	(Gan cao) 10g
		*Paeoniae Radix Alba*	*Bupleuri Radix*	*Aurantii Fructus Immaturus*	
		(Bai shao) 15g	(Chai hu) 25g	(Zhi shi) 10g	
**Zhang 2019**	Y-National Food and Drug Administration National Drug Standards	*Citrus Reticulata*	*Atractylodes macrocephala Koidz*.	*Paeoniae Radix Alba*	*Saposhnikoviae Radix*
		(Chen pi) 15g	(Bai zhu) 25g	(Bai shao) 30g	(Fang feng) 15g
		*Bupleuri Radix*	*Aurantii Fructus*	*Glycyrrhizae Radix et Rhizoma*	*Codonopsis Radix*
		(Chai hu) 15g	(Zhi qiao) 25g	(Gan cao) 10g	(Dang shen) 30g
		*Poria cocos (Schw*.*) Wolf*	*Zingiberis Rhizoma*	*Evodiae Fructus*	
		(Fu ling) 25g	(Gan jiang) 10g	(Wu zhu yu) 6g	
**Zhao et.al 2019**	Y-National Food and Drug Administration National Drug Standards	*Magnolia Officinalis Rehd Et Wils*.	*Rhizoma Dioscoreae*	*Amomum Aurantiacum H*. *T*. *Tsai Et S*. *W*. *Zhao*	*Alpinia Katsumadai Hayat*
		(Hou po) 20g	(Shan yao) 30g	(Sha ren) 10g	(Cao dou kou) 6g
		*Hedysarum Multijugum Maxim*. (Huang qi) 15g	*Bupleuri Radix*	*Saposhnikoviae Radix*	*Aconiti Lateralis Radix Praeparata*
			(Chai hu) 6g	(Fang feng) 6g	(Fu zi) 9g
		*Myristicae Semen*	*Atractylodes macrocephala Koidz*.	*Chaenomeles Sinensis (Thouin) Koehne*	*Zingiberis Rhizoma*
		(Rou dou kou) 20g	(Bai zhu) 10g	(Mu gua) 6g	(Gan jiang) 10g
		*Glycyrrhizae Radix et Rhizoma*			
		(Gan cao) 6g			
**Zheng 2019**	Y-National Food and Drug Administration National Drug Standards	*Codonopsis Radix*	*Bupleuri Radix*	*Schizonepetae Herba*	*Saposhnikoviae Radix*
		(Dang shen) 20g	(Chai hu) 10g	(Jing jie) 5g	(Fang feng) 5g
		*Notopterygii Rhizoma Et Radix* (Qiang huo) 5g	*Radix Angelicae Biseratae*	*Poria cocos (Schw*.*) Wolf*	*Aurantii Fructus*
				(Fu ling) 15g	(Zhi qiao) 10g
			(Du huo) 5g		
		*Platycodon Grandiforus*	*Glycyrrhizae Radix et Rhizoma* (Gan cao) 6g		
		(Jie geng) 10g			
**Chen et.al 2018**	Y-National Food and Drug Administration National Drug Standards	*Atractylodes macrocephala Koidz*.	*Citrus Reticulata*	*Paeoniae Radix Alba*	*Saposhnikoviae Radix*
			(Chen pi) 5g	(Bai shao) 6.7g	(Fang feng) 3.7g
		(Bai zhu) 10g			
**Tang et.al 2018**	Y-National Food and Drug Administration National Drug Standards	*Hedysarum Multijugum Maxim*.	*Atractylodes macrocephala Koidz*.	*Paeoniae Radix Alba*	*Saposhnikoviae Radix*
				(Bai shao) 24g	(Fang feng) 9g
		(Huang qi) 18g	(Bai zhu) 18g		
		*Zingiber officinale Roscoe*	*Myristicae Semen*	*Arum Ternatum Thunb*.	*Aucklandiae Radix*
		(Sheng jiang) 6g	(Rou dou kou) 9g	(Ban xia) 9g	(Mu xiang) 12g
		*Citrus Reticulata*	*Coptidis Rhizoma*	*Glycyrrhizae Radix et Rhizoma*	
		(Chen pi) 9g	(Huang lian) 6g	(Gan cao) 6g	
**Fan et.al 2017**	Y-National Food and Drug Administration National Drug Standards	*Atractylodes macrocephala Koidz*.	*Citrus Reticulata*	*Paeoniae Radix Alba*	*Saposhnikoviae Radix*
		(Bai zhu)	(Chen pi)	(Bai shao)	(Fang feng)
**Wang et.al 2017**	Y-National Food and Drug Administration National Drug Standards	*Atractylodes macrocephala Koidz*.	*Lablab Semen Album*	*Coicis Semen*	*Paeoniae Radix Alba*
		(Bai zhu) 20g	(Bai bian dou) 20g	(Yi yi ren) 20g	(Bai shao) 15
		*Cyperi Rhizoma*	*Myristicae Semen*	*Granati Pericarpium*	*Radix Puerariae*
		(Xiang fu) 15g	(Rou dou kou) 15g	(Shi liu pi) 20g	(Ge gen) 20g
**Zhang 2017**	Y-National Food and Drug Administration National Drug Standards	*Aconiti Lateralis Radix Praeparata*	*Panax Ginseng C*. *A*. *Mey*.	*Zingiberis Rhizoma*	*Glycyrrhizae Radix et Rhizoma*
		(Fu zi)	(Ren shen)	(Gan jiang)	(Gan cao)
		*Myristicae Semen*	*Psoralea corylifolia Linn*.	*Schisandrae Chinensis Fructus*	*Evodiae Fructus*
		(Rou dou kou)	(Bu gu zhi)	(Bei wu wei zi)	(Wu zhu yu)
		*Jujubae Fructus*			
		(Da zao)			
**Chen 2016**	Y-National Food and Drug Administration National Drug Standards	*Psoralea corylifolia Linn*.	*Evodiae Fructus*	*Poria cocos(Schw*.*)Wolf*	*Euryales Semen*
		(Bu gu zhi) 10g	(Wu zhu yu) 5g	(Fu ling) 15g	(Qian shi) 15g
		*Myristicae Semen*	*Schisandrae Chinensis Fructus*	*Poria cocos(Schw*.*)Wolf*	*Rhizoma Dioscoreae*
		(Rou dou kou) 10g	(Bei wu wei zi) 10g	(Fu ling) 15g	(Shan yao) 15g
		*Hedysarum Multijugum Maxim*.	*Portulaca Herba*	*Foeniculi Fructus*	*Zingiberis Rhizoma*
		(Huang qi) 15g	(Ma chi xian) 20g	(Xiao hui xiang) 10g	(Gan jiang) 10g
**Huang et.al 2016**	Y-National Food and Drug Administration National Drug Standards	*Bupleuri Radix*	*Aurantii Fructus*	*Paeoniae Radix Alba*	*Atractylodes macrocephala Koidz*.
		(Chai hu) 9g	(Zhi qiao) 4g	(Bai shao) 15g	(Bai zhu) 10g
		*Citrus Reticulata*	*Saposhnikoviae Radix*	*Rhizoma Dioscoreae*	*Codonopsis Radix*
		(Chen pi) 6g	(Fang feng) 9g	(Shan yao) 15g	(Dang shen) 9g
		*Coicis Semen*	*Agrimonia Eupatoria*		
		(Yi yi ren) 15g	(Xian he cao) 15g		
**Bensoussan et.al 2015**	Y- Australian Therapeutic Goods Administration	*Paeoniae Radix Alba*	*Aurantii Fructus Immaturus*	*Magnolia Officinalis Rehd Et Wils*.	*Citrus Reticulata*
		(Bai shao)	(Zhi shi)	(Hou po)	(Chen pi)
		*Glycyrrhizae Radix et Rhizoma*	*Atractylodes lancea (Thunb*.*) DC*.	*Radix Rhei Et Rhizome*	
		(Gan cao)	(Cang zhu)	(Da huang)	
**Cheng 2015**	Y-National Food and Drug Administration National Drug Standards	*Bupleuri Radix*	*Cyperi Rhizoma*	*Chuanxiong Rhizoma*	*Citrus Reticulata*
		(Chai hu) 9g	(Xiang fu) 20g	(Chuan xiong) 9g	(Chen pi) 12g
		*Paeoniae Radix Alba*	*Glycyrrhizae Radix et Rhizoma*	*Aurantii Fructus*	*Atractylodes macrocephala Koidz*.
		(Bai shao) 20g	(Gan cao) 10g	(Zhi qiao) 15g	(Bai zhu) 15g
		*Saposhnikoviae Radix*	*Citri Fructus*	*Codonopsis Radix*	*Radix Puerariae*
		(Fang feng) 6g	(Xiang yuan) 12g	(Dang shen) 15g	(Ge gen) 20g
		*Artemisiae Scopariae Herba*			
		(Yin chen) 6g			
**Huang 2015**	Y-National Food and Drug Administration National Drug Standards	*Paeoniae Radix Alba*	*Atractylodes macrocephala Koidz*.	*Corydalis Rhizoma*	*Poria cocos (Schw*.*) Wolf*
		(Bai shao) 15g	(Bai zhu) 15g	(Yuan hu) 15g	(Fu ling) 15g
		*Ziziphi Spinosae Semen*	*Jasminum polyanthum Franch*.		
		(Suan zao ren) 10g			
			(Su xin hua) 10g		
**Liang et.al 2015**	Y-National Food and Drug Administration National Drug Standards	*Psoralea corylifolia Linn*.	*Evodiae Fructus*	*Myristicae Semen*	*Schisandrae Chinensis Fructus*
		(Bu gu zhi) 10g	(Wu zhu yu) 10g	(Rou dou kou) 10g	(Bei wu wei zi) 10g
		*Poria cocos (Schw*.*) Wolf*	*Rhizoma Dioscoreae*	*Nelumbinis Plumula*	*Euryales Semen*
		(Fu ling) 15g	(Shan yao) 20g	(Lian zi) 15g	(Qian shi) 20g
**Wei 2015**	Y-National Food and Drug Administration National Drug Standards	*Codonopsis Radix*	*Atractylodes macrocephala Koidz*.	*Poria cocos (Schw*.*) Wolf*	*Lablab Semen Album*
		(Dang shen) 15g	*(*Bai zhu) 15g	(Fu ling) 15g	(Bai bian dou) 20g
		*Platycodon Grandiforus*	*Rhizoma Dioscoreae*	*Amomum Aurantiacum H*. *T*. *Tsai Et S*. *W*. *Zhao*	*Coicis Semen*
		(Jie geng) 6g	(Shan yao) 20g	(Sha ren) 3g	(Yi yi ren) 30g
		*Glycyrrhizae Radix et Rhizoma* (Gan cao) 3g			
**Yan 2015**	Y-National Food and Drug Administration National Drug Standards	*Silktree Albizia Bark*	*Poria cocos(Schw*.*)Wolf*	*Atractylodes macrocephala Koidz*.	*Coicis Semen*
		(He huan pi) 20g	(Fu ling) 30g	(Bai zhu) 15	(Yi yi ren) 20g
		*Angelicae Sinensis Radix*	*Paeoniae Radix Alba*	*Bupleuri Radix*	*Caulis Polygoni Multiflori*
		(Dang gui) 12g	(Bai shao) 20g	(Chai hu) 9g	(Shou wu teng) 15g
		*Glycyrrhizae Radix et Rhizoma*	*Cornus Officinalis Sieb*. *ET Zucc*.	*Cyperi Rhizoma*	*Menthae Herba*
		(Gan cao) 10g	(Shan zhu yu) 12g	(Xiang fu) 20g	(Bo he) 10g
**Chen et.al 2014**	Y-National Food and Drug Administration National Drug Standards	*Paeoniae Radix Alba*	*Atractylodes macrocephala Koidz*.	*Coptidis Rhizoma*	*Evodiae Fructus*
		(Bai shao)	(Bai zhu)	(Huang lian)	(Wu zhu yu)
		*Cimicifugae Rhizoma*	*Silktree Albizia Bark*		
		(Sheng ma)	(He huan pi)		
**Cai et.al 2013**	Y-National Food and Drug Administration National Drug Standards	*Codonopsis Radix*	*Paeoniae Radix Alba*	*Atractylodes macrocephala Koidz*.	*Saposhnikoviae Radix*
		(Dang shen)	(Bai shao)	(Bai zhu)	(Fang feng)
		*Citrus Reticulata*	*Curcumae Radix*	*Silktree Albizia Bark*	*Glycyrrhizae Radix et Rhizoma*
		(Chen pi)	(Yu jin)	(He huan pi)	(Gan cao)
		*Lablab Semen Album*	*Poria cocos (Schw*.*) Wolf*	*Amomum Aurantiacum H*. *T*. *Tsai Et S*. *W*. *Zhao*	*Platycodon Grandiforus*
		(Bai bian dou)	(Fu ling)	(Sha ren)	(Jie geng)
		*Coicis Semen*	*Alpinia Katsumadai Hayat*		
		(Yi ren)	(Cao dou kou)		
**Bian 2011**	Y-National Food and Drug Administration National Drug Standards	*Hedysarum Multijugum Maxim*.	*Atractylodes macrocephala Koidz*.	*Paeoniae Radix Alba*	*Saposhnikoviae Radix*
		(Huang qi)	(Bai zhu)	(Bai shao)	(Fang feng)
		*Citrus Reticulata*	*Zingiberis Rhizoma*		
		(Chen pi)	(Gan jiang)		
**Liang et.al 2009**	Y-National Food and Drug Administration National Drug Standards	*Paeoniae Radix Alba*	*Bupleuri Radix*	*Atractylodes macrocephala* Koidz.	*Citrus Reticulata*
		(Bai shao) 10g	(Chai hu) 10g	(Bai zhu) 15g	(Chen pi) 10g
		*Saposhnikoviae Radix*	*Poria cocos (Schw*.*) Wolf*	*Aucklandiae Radix*	*Pogostemon Cablin (Blanco) Benth*.
		(Fang feng) 10g	(Fu ling) 15g	(Mu xiang) 6g	(Huo xiang) 10g
		*Coicis Semen*	*Glycyrrhizae Radix et Rhizoma*		
		(Yi yi ren) 30g	(Gan cao) 6g		
**Wu 2009**	Y-National Food and Drug Administration National Drug Standards	*Bupleuri Radix*	*Atractylodes macrocephala Koidz*.	*Paeoniae Radix Alba*	*Saposhnikoviae Radix*
		(Chai hu) 15g	(Bai zhu) 15g	(Bai shao) 30g	(Fang feng) 15
		*Citrus Reticulata*	*Hedysarum Multijugum Maxim*.	*Jujubae Fructus*	*Lablab Semen Album*
		(Chen pi) 5g	(Huang qi) 30g	(Da zao) 15g	(Bai bian dou) 30g
		*Poria cocos (Schw*.*) Wolf* (Fu ling) 15g			
**Zhao 2007**	Y-National Food and Drug Administration National Drug Standards	*Pulsatillae Radix*	*Coptidis Rhizoma*	*Phellodendri Chinrnsis* Cortex	*Fraxini Cortex*
		(Bai tou weng) 9g	(Huang lian) 6g	(Huang bo) 6g	(Qin pi) 12g
		*Mongolian Dandelion Herb*	*Portulaca Herba*	*Citrus Reticulata*	*Atractylodes macrocephala Koidz*.
		(Pu gong ying) 18g	(Ma chi xian) 25g	(Chen pi) 6g	(Bai zhu) 9g
		*Paeoniae Radix Alba*	*Saposhnikoviae Radix*	*Aucklandiae Radix*	*Massa Medicata Fermentata*
		(Bai shao) 9g	(Fang feng) 9g	(Mu xiang) 6g	(Shen qu) 12g
		*Sophorae Flavescentis Radix*	*Radix Sanguisorbae*	*Coicis Semen*	*Angelicae Sinensis Radix*
		(Ku shen) 12g	(Di yu) 12g	(Yi yi ren) 15g	(Dang gui) 15g
		*Glycyrrhizae Radix et Rhizoma* (Gan cao) 6g			
**Leung et.al 2006**	Y-National Food and Drug Administration National Drug Standards	*Atractylodes macrocephala Koidz*.	*Hedysarum Multijugum Maxim*.	*Paeoniae Radix Alba*	*Atractylodes lancea (Thunb*.*) DC*.
		(Bai zhu) 15g	(Huang qi) 15g	(Bai shao) 15g	(Cang zhu) 12g
		*Bupleuri Radix*	*Citrus Reticulata*	*Saposhnikoviae Radix*	*Murraya exotica L*
		(Chai hu) 9g	(Chen pi) 9g	(Fang feng) 9g	(Jiu li xiang) 9g
		*Granati Pericarpium*	*Portulaca Herba*	*Coptidis Rhizoma*	
		(Shi liu pi) 9g	(Ma chi xian) 30g	(Huang lian) 6g	

### Quality assessment of included studies

**Fig 3:**
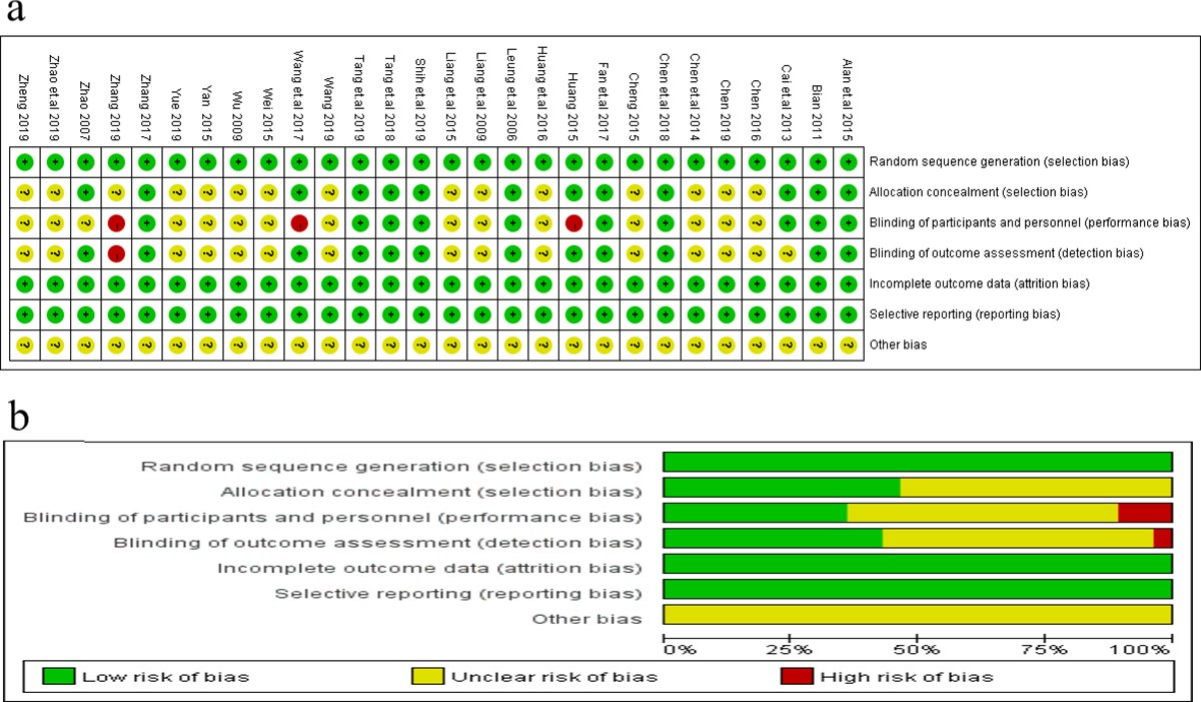
Risk of bias of the included trials: (a) Risk of bias in individual study; (b) Risk of bias summary.

We evaluated the quality of included studies with the Cochrane Collaboration Recommendations assessment tools [53]. All of the studies (28/28) described a random component in the sequence generation process such as a computer-generated random number or a random number table. Allocation concealment was performed using an appropriately sealed method in 17.9% (5/28) of the studies, while 82.1% (23/28) either did not describe concrete methods or used an inappropriate allocation concealment method. In performance bias, 35.7% (10/28) of the included trials reported the methods of blinding for both participants and personnel. In detection bias, 64.3% (18/28) of the outcome assessors in the studies either could not be blinded or were unclear. In attrition bias, all of the studies were deemed to have low-risk outcome data (i.e., reported drop out rates within the range of statistical estimations, provided detailed explanations of drop out rates, or performed intention-to-treat analysis). A detailed quality assessment is presented in Fig 3.

### Primary outcome

#### Adequate Relief (AR)

**Fig 4:**
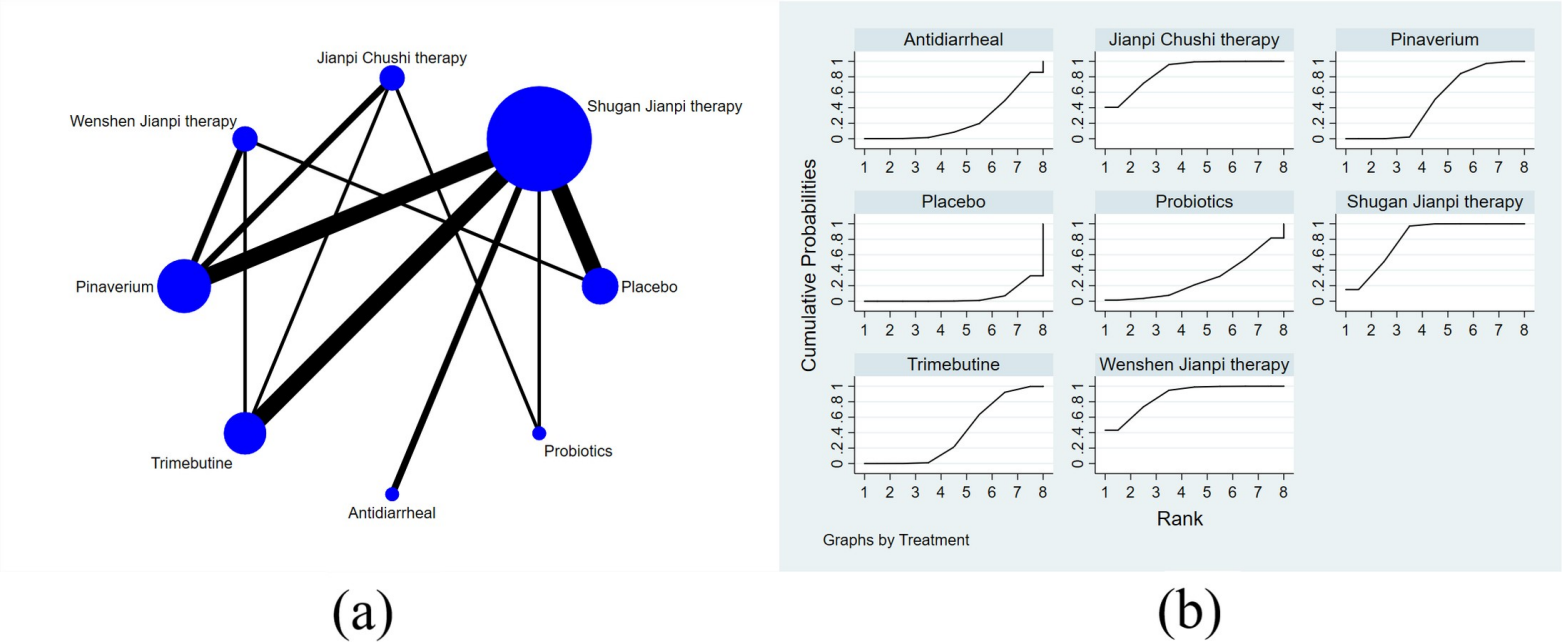
Network meta-analysis of adequate relief: (a) Network evidence plot; (b) Surface under the cumulative ranking curve plot.

**Fig 5:**
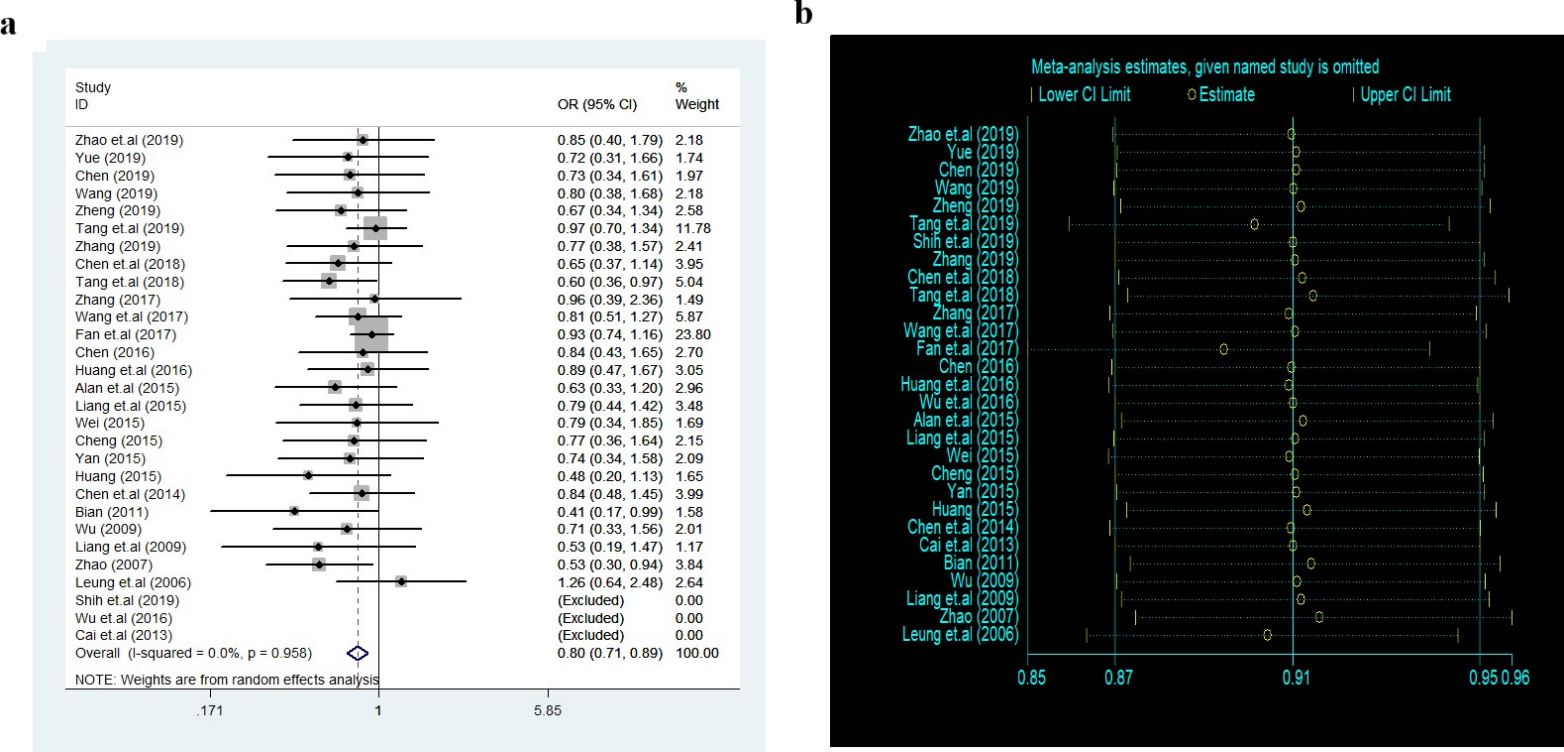
Heterogeneity analysis and sensitivity analysis: (a) Heterogeneity analysis; (b) Sensitivity analysis.

**Fig 6:**
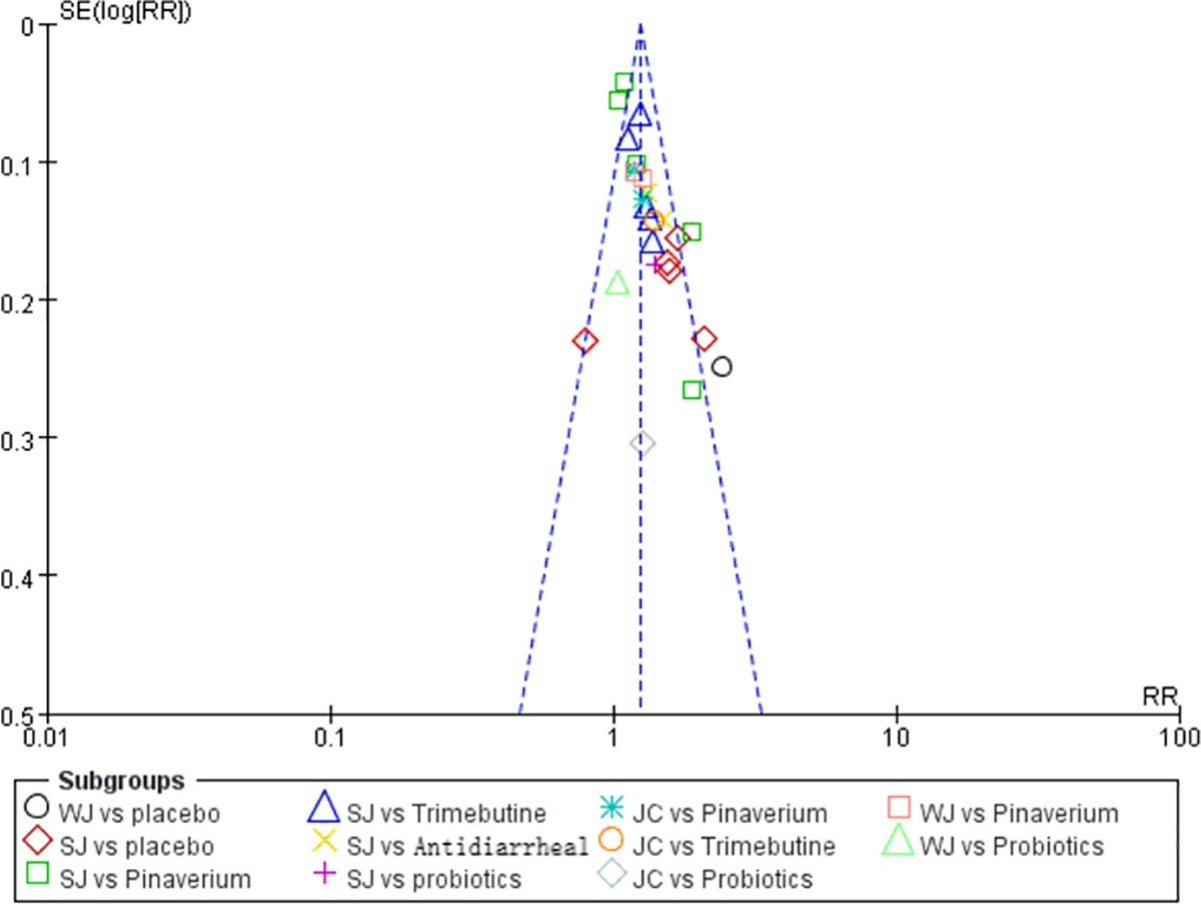
Funnel plot. JC: Jianpi-Chushi therapy; SJ: Shugan-Jianpi therapy; WJ: Wenshen-Jianpi therapy.

A total of 26 studies with 8 treatments reported adequate relief. The specific network is presented in Fig 4A. In terms of efficacy (Table 4), JC was better than the placebo (RR 1.79, 95% CI 1.49 to 2.15), pinaverium (RR 1.28, 95% CI 1.14 to 1.45), trimebutine (RR 1.43, 95% CI 1.24 to 1.64), probiotics (RR 1.54, 95% CI 1.13 to 2.10), antidiarrheal (RR 1.62, 95% CI 1.30 to 2.02) and the differences were statistically significant. The efficacy of WJ (RR 1.70, 95% CI 1.39 to 2.17) and SJ (RR 1.52, 95% CI 1.30 to 1.78) were also better than placebo and rank 2^nd^, 3^rd^ among all the therapies. The treatments were ranked as follow according to the SUCRA (Fig 4B): JC > WJ > SJ > pinaverium > trimebutine > probiotics > antidiarrheal > placebo. The heterogeneity in Fig 5A indicated good homogeneity (*I2* = 0.0%, *P* = 0.958), and sensitivity analysis showed strong stability in Fig 5B. Meanwhile, the symmetry funnel plot was observed in Fig 6.

**Table 4:** Risk ratio with 95% confidence interval of adequate relief.

JC							
1.06 (0.88,1.26)	**WJ**						
**1.18 (1.04,1.34)** *	1.11 (0.96,1.29)	**SJ**					
**1.28 (1.14,1.45)** *	**1.22 (1.06,1.39)** *	**1.09 (1.03,1.16)** *	**Pinaverium**				
**1.43 (1.24,1.64)** *	**1.35 (1.15,1.58)** *	**1.21 (1.12,1.32)** *	**1.11 (1.01,1.23)** *	**Trimebutine**			
**1.54 (1.13,2.10)** *	**1.46 (1.05,2.03)** *	1.31 (0.97,1.76)	1.20 (0.89,1.62)	1.08 (0.79,1.47)	**Probiotics**		
**1.62 (1.30,2.02)** *	**1.53 (1.21,1.93)** *	**1.37 (1.15,1.65)** *	**1.26 (1.04,1.52)** *	1.13 (0.93,1.38)	1.05 (0.74,1.49)	**Antidiarrheal**	
**1.79 (1.49,2.15)** *	**1.70 (1.39,2.07)** *	**1.52 (1.30,1.78)** *	**1.39 (1.18,1.64)** *	**1.26 (1.05,1.50)** *	1.16 (0.83,1.63)	1.11 (0.87,1.41)	**Placebo**

Annotation

*P<0.05. JC: Jianpi-Chushi therapy; WJ: Wenshen-Jianpi therapy; SJ: Shugan-Jianpi therapy.

#### Irritable bowel syndrome—severity scoring system (IBS-SSS)

**Fig 7:**
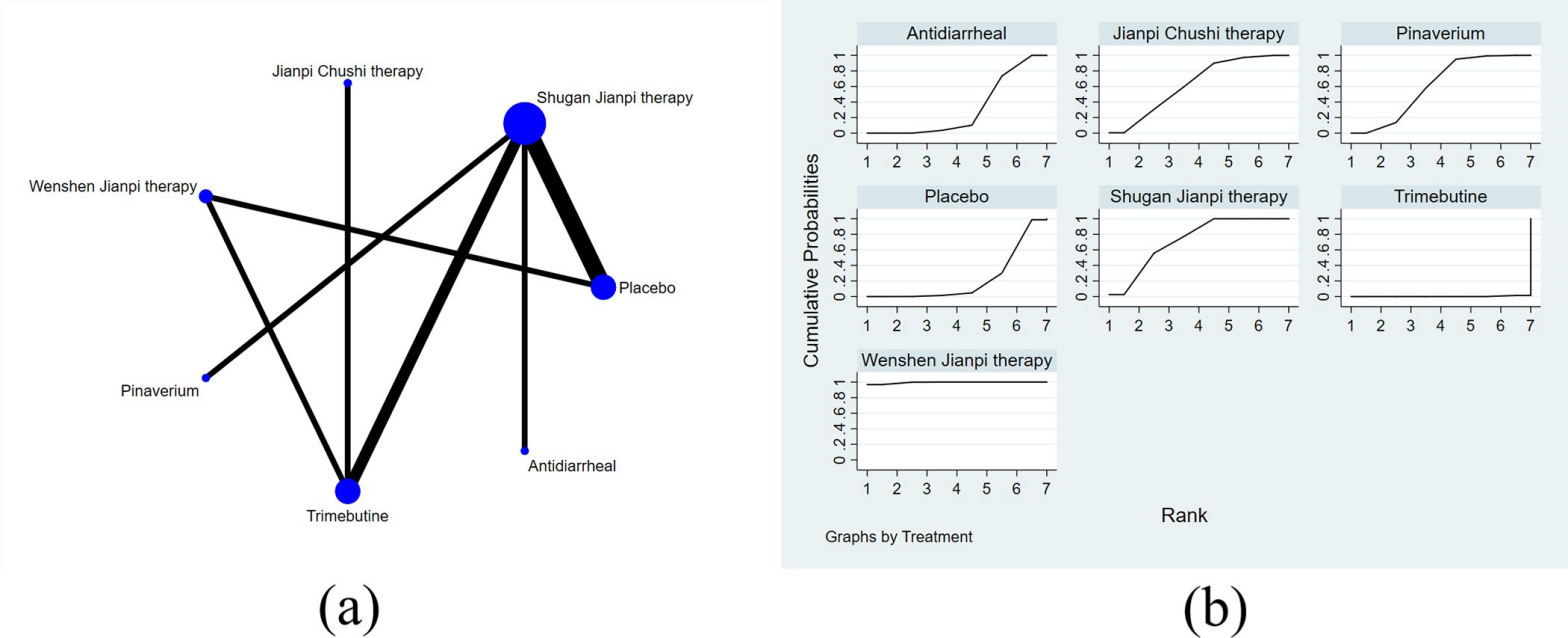
Network meta-analysis of IBS-severity scoring system: (a) Network evidence plot; (b) Surface under the cumulative ranking curve plot.

The improvement of IBS-SSS was reported in 10 studies with 7 treatments. The specific network is presented in Fig 7A. It is revealed from Table 5 that JC was better than trimebutine (SMD 2.93, 95% CI 1.16 to 7.39), antidiarrheal (SMD 14.01, 95% CI 3.42 to 57.50) in the improvement of IBS-SSS. The efficacy of SJ (SMD 8.25, 95% CI 3.34 to 20.35) and WJ (SMD 6.86, 95% CI 2.13 to 22.12) were also better than antidiarrheal. The SUCRA is presented in Fig 7B and the treatments are ranked as follow: JC > SJ > pinaverium > WJ > trimebutine > placebo > antidiarrheal.

**Table 5:** Standard mean difference with 95% confidence interval of irritable bowel syndrome symptom severity scale.

JC						
1.70 (0.57,5.03)	**SJ**					
1.73 (0.46,6.43)	1.02 (0.49,2.13)	**Pinaverium**				
2.04 (0.63,6.63)	1.20 (0.57,2.53)	1.18 (0.41,3.38)	**WJ**			
**2.93 (1.16,7.39)** *	1.72 (0.98,3.02)	1.69 (0.67,4.29)	1.43 (0.69,2.96)	**Trimebutine**		
2.93 (0.93,9.20)	**1.73 (1.09,2.73)** *	1.70 (0.71,4.05)	1.44 (0.70,2.93)	1.00 (0.51,1.96)	**Placebo**	
**14.01 (3.42,57.50)** *	**8.25 (3.34,20.35)** *	**8.11 (2.52,26.07)** *	**6.86 (2.13,22.12)** *	**4.79 (1.65,13.89)** *	**4.78 (1.74,13.17)** *	**Antidiarrheal**

Annotation

*P<0.05. JC: Jianpi-Chushi therapy; WJ: Wenshen-Jianpi therapy; SJ: Shugan-Jianpi therapy.

### Secondary outcome

#### Improvement of clinical symptoms

**Fig 8:**
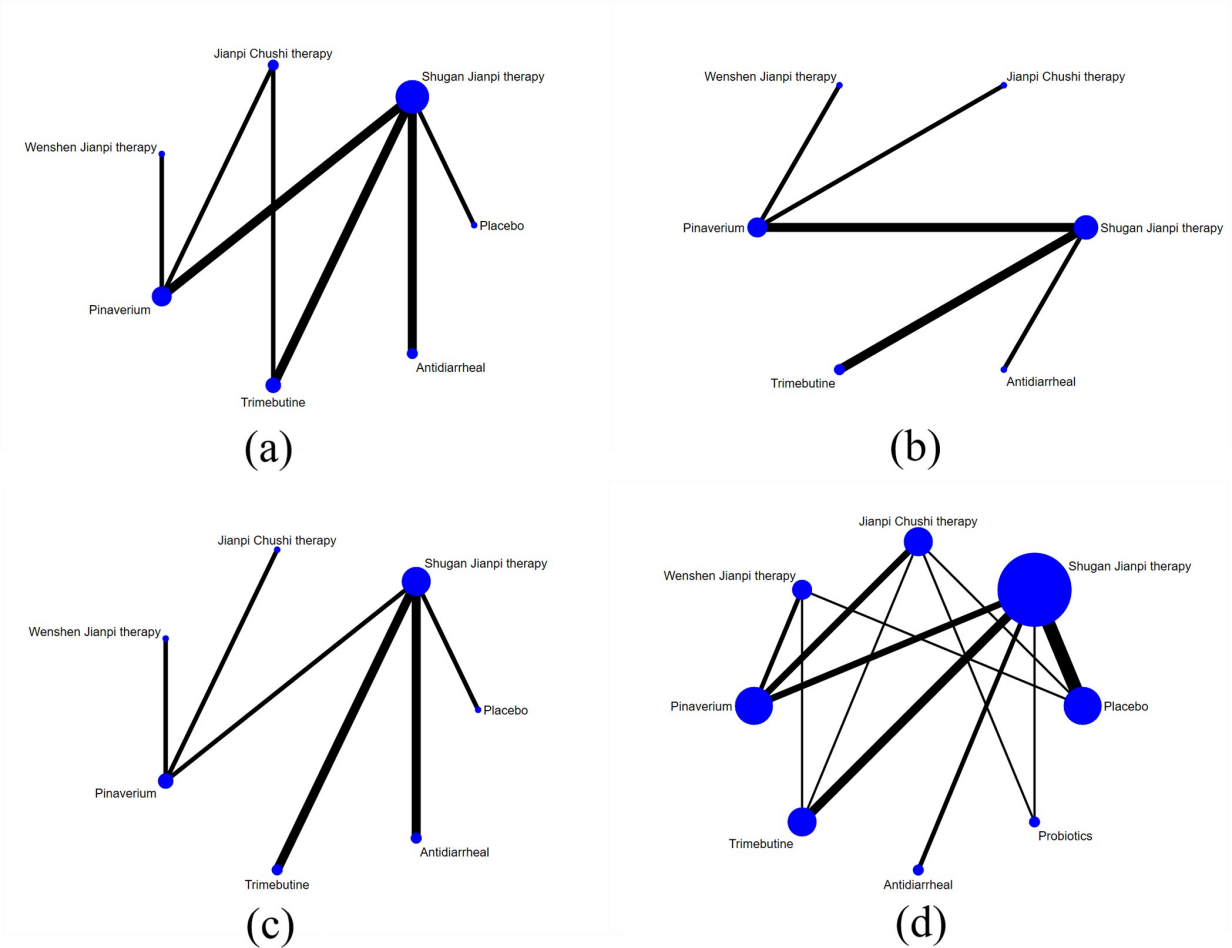
Network evidence of improvement of clinical symptoms and adverse effects: (a) Abdominal pain; (b) Abdominal distension; (c) Stool character; (d) Adverse effects.

This NMA included 3 subgroups: abdominal pain, abdominal distension, and stool character. The specific networks were presented in Fig 8A–8C. As displayed in Table 6, SJ was better than placebo (SMD 1.99, 95% CI 1.04 to 3.83), pinaverium (SMD 1.55, 95% CI 1.01 to 2.40), trimebutine (SMD 1.84, 95% CI 1.25 to 2.70), antidiarrheal (SMD 2.37, 95% CI 1.29 to 4.35) in alleviating abdominal pain. For the abdominal distension, SJ was better than antidiarrheal (SMD 4.01, 95% CI 1.14 to 14.17). As for the stool character, WJ was better than antidiarrheal (SMD 2.79, 95% CI 1.01 to 7.11) and trimebutine (SMD 3.22, 95% CI 1.23 to 8.44). The ranking probabilities of therapies are presented in Fig 10A–10C. Based on the SUCRA for abdominal pain, the therapies are ranked as follow: SJ > JC > WJ > pinaverium > trimebutine > placebo > antidiarrheal; for abdominal distension: SJ > JC > WJ > pinaverium > trimebutine > antidiarrheal; for stool character: WJ > SJ > JC > pinaverium > placebo > antidiarrheal > trimebutine.

**Table 6:** Standard mean difference with 95% confidence interval of clinical improvement.

**Abdominal pain**						
**SJ**						
1.18 (0.67,2.08)	**JC**					
1.51 (0.74,3.09)	1.28 (0.58,2.81)	**WJ**				
**1.55 (1.01,2.40)** *	1.32 (0.77,2.26)	1.03 (0.58,1.83)	**Pinaverium**			
**1.84 (1.25,2.70)** *	1.56 (0.91,2.68)	1.22 (0.56,2.65)	1.18 (0.70,2.00)	**Trimebutine**		
**1.99 (1.04,3.83)** *	1.69 (0.71,4.01)	1.32 (0.50,3.48)	1.28 (0.59,2.81)	1.08(0.51,2.31)	**Placebo**	
**2.37(1.29,4.35)** *	2.01 (0.87,4.61)	1.57 (0.61,4.02)	1.52 (0.72,3.21)	1.29 (0.63,2.65)	1.19 (0.49,2.90)	**Antidiarrheal**
**Abdominal distension**						
**SJ**						
1.34 (0.38,4.73)	**JC**					
1.41 (0.42,4.72)	1.05 (0.25,4.35)	**WJ**				
1.45 (0.71,2.98)	1.08 (0.38,3.05)	1.03 (0.39,2.72)	**Pinaverium**			
1.88 (0.94,3.76)	1.41 (0.33,5.92)	1.34 (0.33,5.38)	1.30 (0.48,3.51)	**Trimebutine**		
**4.01 (1.14,14.17)** *	2.99 (0.50,17.81)	2.85 (0.50,16.33)	2.76 (0.65,11.80)	2.13 (0.51,8.98)	**Antidiarrheal**	
**Stool character**						
**WJ**						
1.17 (0.49,2.82)	**SJ**					
1.34 (0.61,2.92)	1.14 (0.46,2.80)	**JC**				
1.36 (0.80,2.33)	1.16 (0.58,2.33)	1.02 (0.58,1.81)	**Pinaverium**			
2.72 (0.91,8.12)	**2.32 (1.21,4.45)** *	2.03 (0.67,6.18)	1.99 (0.77,5.18)	**Placebo**		
**2.79 (1.01,7.71)** *	**2.37 (1.42,3.97)** *	2.08 (0.74,5.88)	2.04 (0.86,4.86)	1.03 (0.45,2.35)	**Antidiarrheal**	
**3.22 (1.23,8.44)** *	**2.75 (1.85,4.08)** *	2.41 (0.90,6.44)	**2.36 (1.06,5.26)** *	1.19 (0.55,2.54)	1.16 (0.60,2.22)	**Trimebutine**

Annotation

*P<0.05. JC: Jianpi-Chushi therapy; WJ: Wenshen-Jianpi therapy; SJ: Shugan-Jianpi therapy.

#### Adverse effects

**Fig 9:**
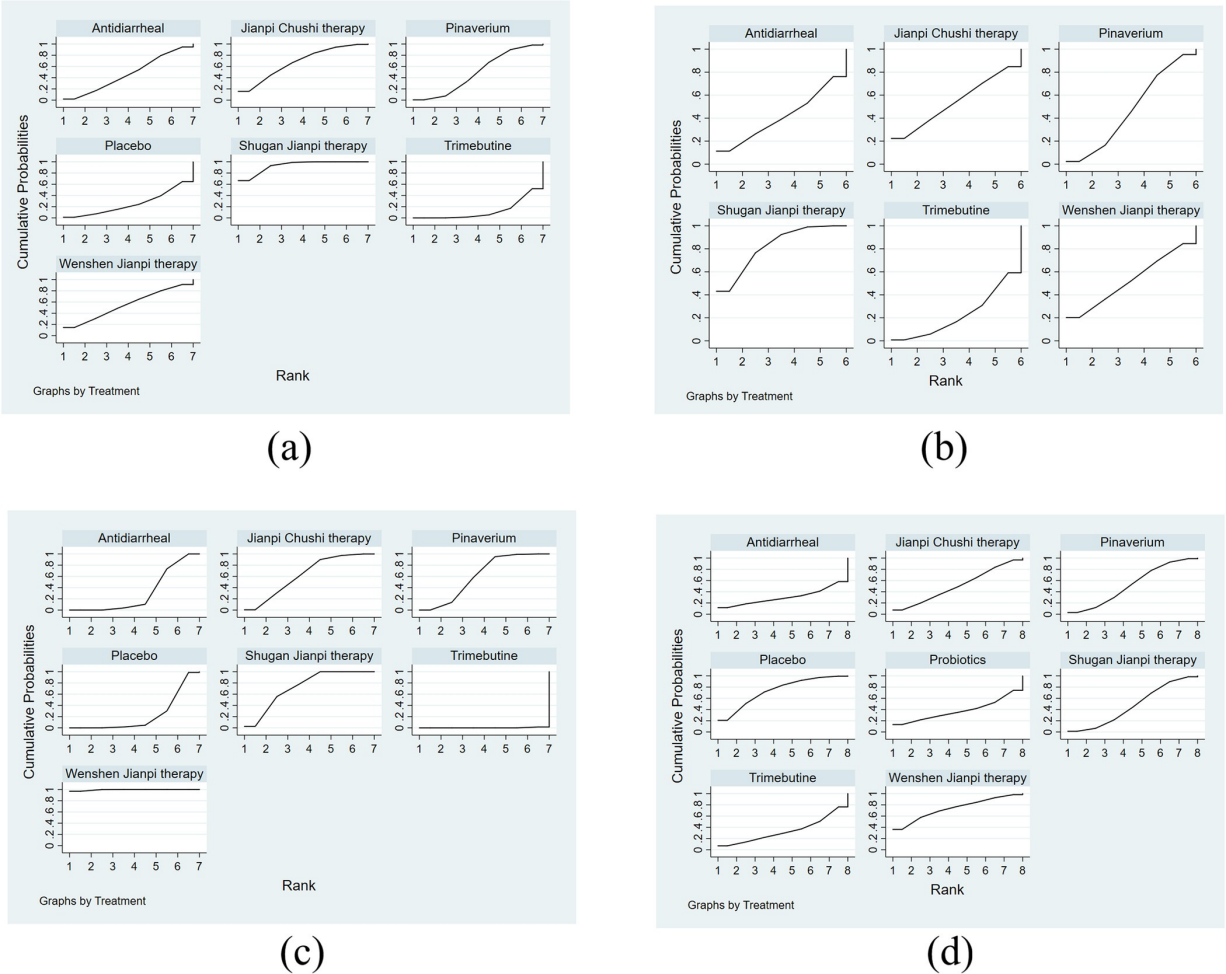
Surface under the cumulative ranking curve plot: (a) Abdominal pain; (b) Abdominal distension; (c) Stool character; (d) Adverse effects.

There were 26 studies with 8 treatments that reported adverse effects. The most common side effects in the treatment groups were nausea and vomiting, constipation, and slight elevation of liver aminotransferases while abdominal pain and distension, nausea, and flatulence in the controlled groups. The specific network was presented in Fig 8D. The result in Table 7 indicated that there were no significant statistical differences among all the therapies. Based on the SUCRA in Fig 9D, the treatments are ranked as follow: WJ > placebo > Pinaverium > JC > SJ > Probiotics > Trimebutine > Antidiarrheal.

**Table 7:** Risk ratio with 95% confidence interval of adverse effects.

WJ							
0.91 (0.32,2.64)	**Placebo**						
0.68 (0.21,2.21)	0.74 (0.35,1.57)	**Pinaverium**					
0.66 (0.17,2.60)	0.72 (0.27,1.96)	0.97 (0.41,2.31)	**JC**				
0.65 (0.20,2.08)	0.71 (0.35,1.45)	0.96 (0.72,1.28)	0.98(0.41,2.38)	**SJ**			
0.48 (0.06,3.70)	0.52 (0.08,3.23)	0.70 (0.12,4.03)	0.72(0.15,3.55)	0.73 (0.13,4.22)	**Probiotics**		
0.46 (0.10,2.13)	0.51 (0.12,2.16)	0.68 (0.17,2.73)	0.70(0.15,3.29)	0.71 (0.18,2.79)	0.97 (0.11,8.50)	**Trimebutine**	
0.37 (0.04,3.84)	0.41 (0.05,3.47)	0.55 (0.07,4.24)	0.57(0.06,5.13)	0.58 (0.08,4.33)	0.79 (0.05,11.34)	0.81 (0.07,9.27)	**Antidiarrheic**

Annotation

*P<0.05. JC: Jianpi-Chushi therapy; WJ: Wenshen-Jianpi therapy; SJ: Shugan-Jianpi therapy.

#### GRADE quality evidence

**Fig 10:**
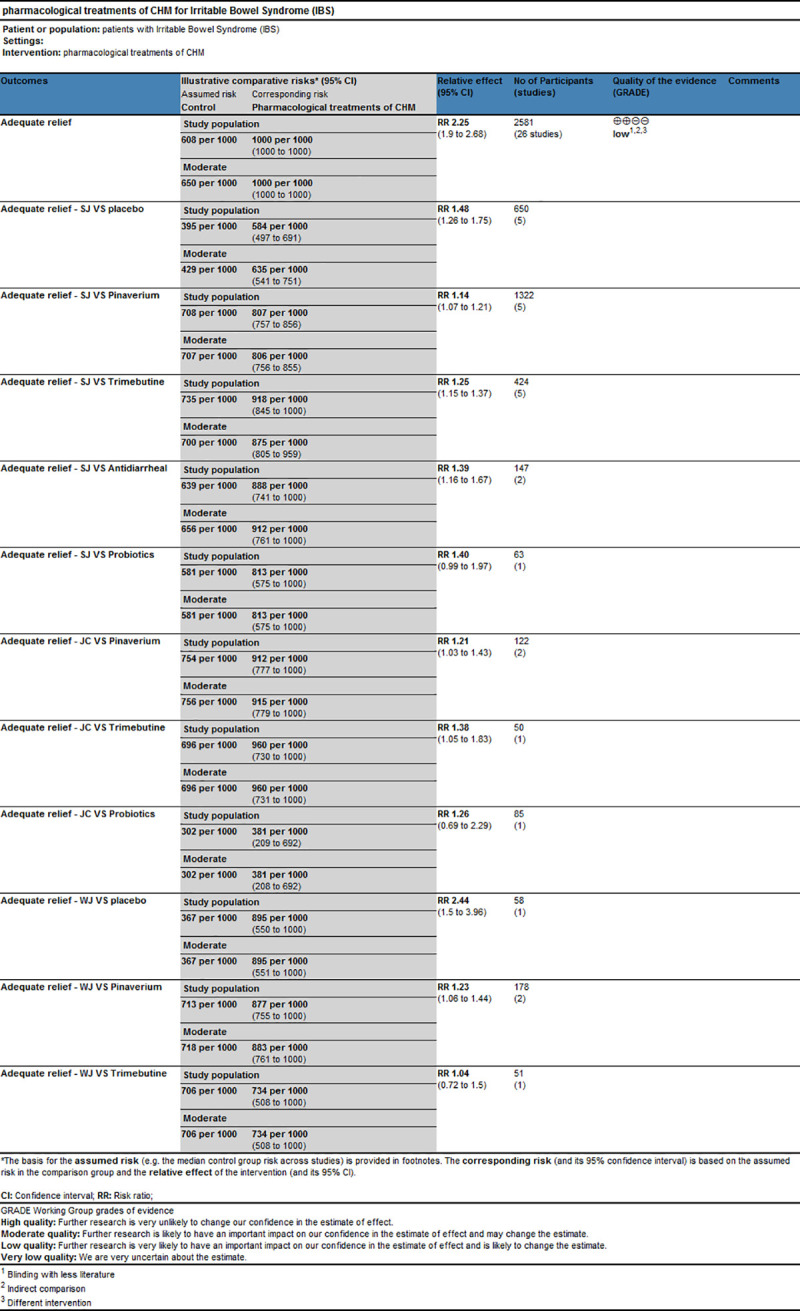
Grading of recommendations assessment, development and evaluation quality grading assessment.

The application of the GRADE approach aims to provide ratings for the confidence in the estimates of effect for specific comparison [54]. There are five elements to downgrade the quality of evidence: risk of bias, inconsistency, indirectness, imprecision, and publication bias while three factors to upgrade: large effect, plausible confounding that would change effect, dose-response gradient. Based on these criteria, the evidence quality of adequate relief was “low”, which could be attributed to the high risk of bias and indirectness. The result of the GRADE assessment was presented in Fig 10.

## Discussion

The treatments of IBS are largely based on its subtypes [1, 55]. In this study, there were 26 trials focus on IBS-D [25, 27–39, 41–54], 1 trial on IBS-C [40], and 1 trial on IBS (contained both IBS-D and IBS-C) [26]. Limited by the treatments of the controlled group, we could only compare TCM with placebo, pinaverium, trimebutine, antidiarrheal, and probiotics. Pinaverium, trimebutine, and probiotics are universal therapies for all types of IBS in relieving abdominal pain while antidiarrheal suits patients with IBS-D.

This NMA systematically evaluated the AR, improvement of IBS-SSS, the improvement of clinical symptoms, and adverse effects after the application of CHM as compared to conventional pharmacological therapies for patients with IBS. In patients with IBS-D, JC performed the best in AR and the improvement of IBS-SSS compared with placebo and any other pharmacological treatments. WJ showed great improvement in improving stool character. SJ had better effects on relieving abdominal pain and abdominal distension. Similarity, in patients with IBS-C, JC also was more effective on adequate relief and in improving stool consistency compared to placebo [40]. There was no difference between CHM and other therapies in adverse effects. In conclusion, CHM could be more beneficial to patients with IBS in decreasing their clinical symptoms and improving their quality of life, which provided more suggestions and guidance in clinical decisions.

As is mentioned before, the etiologies of IBS are diverse, so it is hard to treat it from one single dimension. The core principle of diagnosing and treating disease in TCM is a treatment based on syndrome differentiation (TCM jargon: bian zheng lun zhi”) [56]. According to TCM theory, syndrome (TCM jargon: Zheng) is a presentation of the pathological changes of a certain disease course, revealing the location, cause, and nature of a disease, the correlation between pathogenic factors and health factors, and the body’s ability to resist disease, and thus is a precondition and fundamental for diagnosis and treatments [57]. Under the principle of “bian zheng lun zhi”, the CHM formulae, composed of many different herbs, take the basic prescription as the core and add or delete some drugs on the condition of patients’ symptoms. Therefore, the effective substance of CHM formulae is multi-component, and its functions are multi-target, multi-pathway, and multi-effects. A review study involved 67784 IBS participants found out that the major syndromes of IBS patients were the syndrome of liver stagnation and spleen deficiency, spleen-stomach weakness, and spleen-kidney yang deficiency [58]. Therefore, based on the syndrome differentiation, the treatment of CHM formulae was concluded as Shugan-Jianpi therapy, Jianpi-Chushi therapy, and Wenshen Jianpi therapy.

Shugan-Jianpi therapy mainly consists of herbs such as *Atractylodes macrocephala Koidz*. (Bai zhu), *Bupleuri Radix (*Chai hu*)*, *Paeoniae Radix Alba* (Bai shao), which can influence the expression of transient receptor potential vanilloid-1 and Calcitonin Gene-Related Peptide (CGRP) in the colon tissue of the rat model with visceral hypersensitivity by increasing the pressure threshold of abdominal inwards reflex affected by colorectal distension so that to decrease the visceral sensitivity [59]. Besides, a clinical trial showed that Shugan therapy can also regulate the IBS-D patients’ immune system by decreasing the number of IgM in the serum while enhancing the transformation of T-lymphocyte and increasing the number of T8 + lymphocyte [60]. Jianpi-Chushi therapy, which mostly contains herbs such as *Atractylodes macrocephala Koidz*. (Bai zhu), *Citrus Reticulata* (Chen pi), *Poria Cocos (Schw*.*)* Wolf (Fu ling), can regulate the intestinal flora by reducing the number of aerobes as well as increasing the probiotics, which can significantly relieve the clinical symptoms and achieve ideal effect [61]. Wenshen-Jianpi therapy, largely consists of *Myristicae Semen* (Rou dou kou), Z*ingiberis Rhizoma* (Gan jiang), can regulate the expression of gastrointestinal hormones and their receptors such as melatonin, cholecystokinin, and CGRP [62]. Another clinical study also found that Wenshen-Jianpi therapy can regulate the expression of neurotransmitters such as 5-HT, neuropeptide Y, and immune factors such as TNF-γ [63]. In conclusion, CHM formulae can act on the IBS patients through multi-targets and multi-pathway, so that to improve their clinical symptoms There were several limitations to this meta-analysis. Firstly, the quality of included trials was not optimal due to methodological shortcomings. When evaluating these studies, we found that many lacked details on allocation concealment or blinding. The former will cause selection bias and the latter will result in detection bias. Besides, although many studies reported the dropouts, only 3 studies [34, 41, 44] performed intention-to-treat (ITT), which, to some extent, may lead to incomplete outcome data and increase the attrition bias. Secondly, most of the included trials were single center with only 5 multicenter studies [27, 33, 34, 35, 40] and small sample sizes. The study contains 20 arms of SJ with 1361 patients, but there were only 4 arms of JC with 130 patients and 4 arms of WJ involving 163 patients. Due to the limited number of trials, the results of JC and WJ may cause bias. Therefore, more multi-center and large-scale trials should be conducted to offer more proofs in the future. Thirdly, the diversity of different CHM formulae may generate heterogeneity. Although we classified CHM formulae into 3 categories based on their function, the constitution of herbs was different from one formula to another and the dosage of each formula was personalized. Therefore, the differentiation of herbs and ingredients may affect the final effects. Moreover, the variation in the herbs themselves such as source, preparation, complication proportion, and decoction time might all be the source of heterogeneous. Besides, the differentiation of Chinese medicine formulations such as decoction, capsules, and powder, may influence the chemical composition and may result in heterogeneous. Fourthly, nearly all of the included trials were conducted in China and the populations were Chinese, which will generate publication and cultural bias. In addition, the positive-controlled in this study were not strictly in accord with the guideline. Hence, it does limit the value of the evidence, and more clinical trials using standard treatments as a comparison should be conducted in the future. Further, most of our included studies involved patients with IBS-D, which makes it hard to evaluate the efficacy of TCM in other subtypes of IBS. Finally, the treatment course of the included studies varied from 4 to 8 weeks, most of which lack long-term follow-up. Consequently, the recurrent rate remained unclear after treatment and thus was unable to evaluate the long-term efficacy of CHM formulae. In conclusion, it is still hard to find out whether patients with IBS in large-scale trials and other races can still get similar benefits from CHM formulae in the long-term use.

## Conclusion

Evidence from this NMA confirmed that Shugan-Jianpi therapy, Jianpi-Chushi therapy, and Wenshen-Jianpi therapy could be beneficial for patients with IBS in relieving their different dimensions of clinical symptoms and improving their quality of life. These findings could provide physicians and patients with appropriate treatments based on the specific characteristics of IBS. However, additional high-quality RCTs should be performed to provide more powerful evidence in a wider population of IBS patients.

## Supporting information

S1 File PRISMA checklist. (PDF)

S2 File Search strategy. (PDF)

S3 File Access to include trials. (PDF)
