# Supplementary material for: Pharmacological treatments of Chinese herbal medicine for irritable bowel syndrome in adults: A network meta-analysis of randomized controlled trials
Source: PLoS One. 2021 Aug 6;16(8):e0255665. doi: 10.1371/journal.pone.0255665 (PMC8345858; doi:10.1371/journal.pone.0255665)
Supplement: S2 File — (PDF) [file pone.0255665.s002.pdf]

Search strategies of each database

PubMed:

#1 irritable bowel syndrome [SH]

#2 IBS [SH]

#3 irritable bowel disease [SH]

#4 functional bowel disease [SH]

#5 exp irritable colitis [SH]

#6 exp allergic colitis [SH]

#7 exp colon allergy [SH]

#8 #1 or #2 or #3 or #4 or #5 or #6 or #7

#9 exp traditional Chinese medicine [SH]

#10 exp Chinese medicinal herb [SH]

#11 exp traditional Chinese herbal formula [SH]

#12 exp herbs [SH]

#13 #9 or #10 or #11 or #12

#14 randomized controlled trial [PT]

#15 controlled clinical trial [PT]

#16 randomized [TIAB]

#17 randomly [TIAB]

#18 trial [TIAB]

#19 #14 or #15 or #16 or #17 or #18

#20 #8 and #13 and #19

Annotation: Mesh Subheadings [SH]; Publication Type [PT]; Title/Abstract [TIAB]

Springer Link:

#1 traditional Chinese medicine OR Chinese medicinal herb OR traditional Chinese herbal formula OR herbs

#2 irritable bowel syndrome OR IBS OR irritable bowel disease OR irritable colitis OR functional bowel disease OR allergic colitis OR colon allergy

#3 randomized controlled trial OR controlled clinical trial OR randomized

#4 #1 AND #2 AND #3

EMBASE:

1 (irritable bowel syndrome or IBS or irritable bowel disease or irritable colitis).af.

2 exp functional bowel disease/ or exp allergic colitis/ or exp colon allergy/

3 1 or 2

4 (traditional Chinese medicine).af.

5 exp Chinese medicinal herb/ or exp traditional Chinese herbal formula/ or exp herbs/

6 4 or 5

7 (random* OR factorial* OR crossover*).af.

8 exp crossover-procedure/ or exp double-blind procedure/ or exp randomized controlled trial/ or single-blind procedure/

9 7 or 8

10 3 and 6 and 9

CNKI (China National Knowledge Infrastructure):

#1 traditional Chinese medicine

#2 irritable bowel syndrome OR IBS

#3 randomized controlled trial OR controlled clinical trial OR randomized

#4 #1 AND #2 AND #3

CBM (Chinese Biomedicine Database)

#1 traditional Chinese medicine

#2 irritable bowel syndrome OR IBS

#3 randomized controlled trial OR controlled clinical trial OR randomized

#4 #1 AND #2 AND #3

Wanfang:

#1 traditional Chinese medicine

#2 irritable bowel syndrome OR IBS

#3 randomized controlled trial OR controlled clinical trial OR randomized

#4 #1 AND #2 AND #3
