# Supplementary material for: Pharmacological treatments of Chinese herbal medicine for irritable bowel syndrome in adults: A network meta-analysis of randomized controlled trials
Source: PLoS One. 2021 Aug 6;16(8):e0255665. doi: 10.1371/journal.pone.0255665 (PMC8345858; doi:10.1371/journal.pone.0255665)
Supplement: S3 File — (PDF) [file pone.0255665.s003.pdf]

1. Chen C. A clinical observation on the treatment of Diarrhea-Predominant Irritable Bowel Syndrome (Liver depression and Spleen deficiency pattern) with modified Tongxie Formula: Hunan University of Chinese Medicine; 2019.
Availability: http://doi.org/10.27138/d.cnki.ghuzc.2019.000149

2. Shih Y, Tsai C, Li T, Lai H, Wang K, Liao W, et al. The effect of Xiang-Sha-Liu-Jun-Zi tang (XSLJZT) on irritable bowel syndrome: A randomized, double-blind, placebo-controlled trial. J Ethnopharmacol. 2019; 238:111889.
Availability: http://doi.org/10.1016/j.jep.2019.111889

3. Tang XD, Zhang SS, Hou XH, Li ZH, Chen SN, Feng PM, et al. Post-marketing Re-evaluation of Tongxiening Granules in Treatment of Diarrhea-Predominant Irritable Bowel Syndrome: A Multi-center, Randomized, Double-Blind, Double-Dummy and Positive Control Trial. Chin J Integr Med. 2019;25(12):887-94.
Availability: http://doi.org/10.1007/s11655-019-3030-x

4. Wang X. Clinical observation on treatment of diarrhea-predominant irritable bowel syndrome (spleen and stomach weakness type) with modified YiGong Decoction: Hubei University of Traditional Chinese

Medicine; 2019.
Availability:
http://kns.cnki.net/KCMS/detail/detail.aspx?FileName=1019111331.nh&DbName=CMFD2019

5. Yue Z. A clinical observation on Gegenqinlian Decoction with Sini Decoction in the treatment of IBS-D (Dampness-Heat pattern): Guangxi University of Chinese Medicine; 2019.
Availability: http://doi.org/10.27879/d.cnki.ggxzy.2019.000156

6. Zhang S. Research on Diarrhea formula in treating diarrhea-predominant irritable bowel syndrome with syndrome of Liver depression and Spleen deficiency: Chengdu University of TCM; 2019.
Availability: http://doi.org/10.26988/d.cnki.gcdzu.2019.000467

7. Zhao H, Wu X, Yang Y, Ge Z. Therapeutic Evaluation of Irritable Bowel Syndrome with Diarrhea Treated by Warming the Spleen and Stomach and Nourishing the Gut. Chinese General Practice. 2019;22(25):3137-43.
Availability:
http://kns.cnki.net/KCMS/detail/detail.aspx?FileName=QKYX201925026&DbName=CJFQ2019

8. Zheng F. Clinical observation on Ren Shen Bai Du powder modification treating diarrhea-predominant irritable bowel syndrome (Liver-stagnation and Spleen-deficiency syndrome): Chengdu University of CHM; 2019.

Availability: http://doi.org/10.26988/d.cnki.gcdzu.2019.000610

9. Chen M, Tang TC, Wang Y, Shui J, Xiao XH, Lan X, et al. Randomised clinical trial: Tong-Xie-Yao-Fang granules versus placebo for patients with diarrhoea-predominant irritable bowel syndrome. Aliment Pharm Ther. 2018;48(2):160-8.

Availability: http://doi.org/10.1111/apt.14817

10. Tang X, Lu B, Li Z, Wei W, Meng L, Li B, et al. Therapeutic Effect of Chang’an I Recipe (肠安 Ⅰ 号方) on irritable bowel syndrome with Diarrhea: A Multicenter Randomized Double-Blind Placebo-Controlled Clinical Trial. Chin J Integr Med. 2018;24(9):645-52.

Availability: http://doi.org/10.1007/s11655-016-2596-9

11. Wang JD, Yang ZT, Qiu XP, Niu KM, Shen QY, T Z. Observation of the therapeutic effects of methods of liver-dispersing. spleen-invigorating, kidney-reinforcing and intestine-strengthening on diarrhea-

predominant irritable bowel syndrome. Beijing Journal of Traditional Chinese Medicine. 2017;36(08):696-9.
Availability: http://doi.org/10.16025/j.1674-1307.2017.08.009

12. Fan H, Zheng L, Lai Y, Lu W, Yan Z, Xiao Q, et al. Tongxie Formula Reduces Symptoms of Irritable Bowel Syndrome. Clin Gastroenterol H. 2017;15(11):1724-32.
Availability: http://doi.org/10.1016/j.cgh.2017.06.026

13. Zhang Y. A clinical observation on Treatment of Diarrhea Predominant Irritable Bowel Syndrome with different syndrome types of Traditional Chinese Medicine Granules.: Hubei University of Traditional Chinese Medicine; 2017.
Availability:
http://kns.cnki.net/KCMS/detail/detail.aspx?FileName=1017093828.nh&DbName=CMFD2018

14. Chen M. Observation of the Curative Effect of BaShen Decoction in the Treatment of Diarrhea Irritable Bowel Syndrome of Spleen and Kidney Yang Deficiency.: Fujian University of Traditional Chinese Medicine; 2016.
Availability:

http://kns.cnki.net/KCMS/detail/detail.aspx?FileName=1016224371.nh&DbName=CMFD2017

15. Hang M, Chen Q, Huang J, Li S, Wang W, Wang X, et al. Observation of curative effect of the method of regulating the function of liver and spleen on diarrhea-predominate irritable bowel syndrome. Modern Journal of Integrated Traditional Chinese and Western Medicine. 2016;25(22):2406-7.
Availability: http://kns.cnki.net/KCMS/detail/detail.aspx?FileName=XDJH201622005&DbName=CJFQ2016

16. Bensoussan A, Kellow JE, Bourchier SJ, Fahey P, Shim L, Malcolm A, et al. Efficacy of a Chinese Herbal Medicine in Providing Adequate Relief of Constipation-predominant irritable bowel syndrome: A Randomized Controlled Trial. Clin Gastroenterol Hepatol. 2015;13(11):1946-54.
Availability: http://doi.org/10.1016/j.cgh.2015.06.022

17. Cheng Y. The clinical study of Chaishao Tiaogan Decoction treatment on Diarrhea-Predominant Irritable Bowel Syndrome of liver stagnation and spleen deficiency type: Hebei Medical University; 2015.

Availability:
http://kns.cnki.net/KCMS/detail/detail.aspx?FileName=1015325778.nh&DbName=CMFD2016

18. Huang S. The study of Efficacy and mechanism in IBS-D' Treatment by ChangJiLing based on the "Three Blood Organs" Theory.: Guangzhou University of Chinese Medicine; 2015.
Availability:
http://kns.cnki.net/KCMS/detail/detail.aspx?FileName=1016279502.nh&DbName=CDFD2017

19. Liang H, Chen Q, Wu Y, Chen Y, Huang D. Clinical observation of Bashen Decoction in the treatment of Diarrhea-predominant irritable bowel syndrome. Traditional Chinese Medicine Journal. 2015;14(03):60-3.
Availability: http://doi.org/10.14046/j.cnki.zyytb2002.2015.03.027

20. Wei D. Clinical Research of Shenling Baizhu Decoction combined with Pinaverium and Probiotics in the treatment of Diarrhea-Predominant Irritable Bowel Syndrome (Spleen deficiency with dampness encumbrance).: Zhejiang Chinese Medical University; 2015.
Availability:

http://kns.cnki.net/KCMS/detail/detail.aspx?FileName=1015650423.nh&DbName=CMFD2016

21. Yan D. The clinical study of Hehuanlingzhu Decoction treatment on Diarrhea-predominant irritable bowel syndrome of Liver qi stagnation and mind dystrophy type.: Hebei Medical University; 2015. Availability: http://kns.cnki.net/KCMS/detail/detail.aspx?FileName=1015325780.nh&DbName=CMFD2016

22. Chen M, Chen J, Xia L, Fu R, Lu Z. reating irritable bowel syndrome with Diarrhea Patients by Yigan Fupi Decoction: A Randomized Controlled Trial. Chinese Journal of Integrated Traditional and Western Medicine. 2014;34(06):656-60. Availability: http://kns.cnki.net/KCMS/detail/detail.aspx?FileName=ZZXJ201406004&DbName=CJFQ2014

23. Cai L, Lv B, Meng L, Ma L, Fan Y. Efficacy of Patients with Diarrhea-Predominant Irritable Bowel Syndrome Treated with Treatment of Dispersing Stagnated Liver-Qi Invigorating Spleen and Warming Kidney. Chinese Archives of Traditional Chinese Medicine.

2013;31(05):1097-9.

Availability: http://doi.org/10.13193/j.archtcm.2013.05.139.cailj.026

24. Bian L. Clinical effect evaluation of Chang'anyihao Decoction in treating IBS-D and Research of IBS clinical effect assessment indices.: China Academy of Chinese Medical Sciences; 2011.

Availability:

http://kns.cnki.net/KCMS/detail/detail.aspx?FileName=1011201601.nh&DbName=CDFD2011

25. Liang Z, Chen R, Xu Y, Chen Q, Dong M. Tiaohe Ganpi Hexin Decoction in the treatment of irritable bowel syndrome with diarrhea: a randomized controlled trial. Journal of Integrative Medicine. 2009;7(09):819-22.

Availability:

http://kns.cnki.net/KCMS/detail/detail.aspx?FileName=XBZX200909004&DbName=CJFQ2009

26. Wu W. A clinical study of modified Heganpiyin in the treatment of IBSD.: Guangzhou University of Chinese Medicine; 2009.

Availability:

http://kns.cnki.net/KCMS/detail/detail.aspx?FileName=2009120371.nh&

DbName=CMFD2009

27. Zhao Y. The Experimental and Clinical Study on the Effect of JianpiJieduHuazhuofang on Irritable Bowel Syndrome.: Hebei Medical University; 2007.
Availability:
http://kns.cnki.net/KCMS/detail/detail.aspx?FileName=2007177327.nh&DbName=CDFD2007

28. Leung WK, Wu JC, Liang SM, Chan LS, Chan FK, Xie H, et al. Treatment of Diarrhea-Predominant Irritable Bowel Syndrome with Traditional Chinese Herbal Medicine: A Randomized Placebo-Controlled Trial. The American Journal of Gastroenterology. 2006;101(7):1574-80. Availability: http://doi.org/10.1111/j.1572-0241.2006.00576.x
